# *APOE* Genotype and Statin Response: Evidence From the UK Biobank and All of Us Program

**DOI:** 10.1111/cts.70314

**Published:** 2025-08-05

**Authors:** Innocent G. Asiimwe, Andrea L. Jorgensen, Munir Pirmohamed, the Multimorbidity Mechanism and Therapeutic Research Collaborative (MMTRC), Adam Butterworth, Alasdair Warwick, Alba Fernandez‐Sanles, Albert Henry, Alvina G. Lai, Amanda Roberts, Ana Torralbo, Anoop D. Shah, Aroon Hingorani, Arturo Gonzalez‐Izquierdo, Maria Carolina Borges, Caroline Dale, Chris Finan, Claudia Langenberg, Daniel C. Alexander, Deborah Lawlor, Diana Dunca, Eda B. Ozyigit, Amand F. Schmidt, Harry Hemingway, Honghan Wu, Jasmine Gratton, Jean Gallagher, Jorgen E. Engmann, Lauren E. Walker, Victoria L. Wright, Magdalena Zwierzyna, Margaret Ogden, Martin Cox, Mary Mancini, Michail Katsoulis, Mira Hidajat, Natalie Fitzpatrick, Nishi Chaturvedi, Rashmi Kumar, Rohan Takhar, Sandesh Chopade, Simon Ball, Spiros Denaxas, Tina Shah, Valerie Kuan, Nikita Hukerikar, Reecha Sofat, Frances Bennett, David Ryan, Maik Pietzner

**Affiliations:** ^1^ Department of Pharmacology and Therapeutics, Institute of Systems, Molecular and Integrative Biology University of Liverpool Liverpool UK; ^2^ Department of Health Data Science, Institute of Population Health Sciences University of Liverpool Liverpool UK

**Keywords:** All of Us Research Program, *APOE* genotype, lipid response, major adverse cardiovascular events, mortality, statins, UK Biobank

## Abstract

*APOE* genotype may affect statin response. Using UK Biobank (UKB) and All of Us (AoU) data, we aimed to investigate associations between *APOE* genotype, statin use, and key health outcomes. Our analysis included UKB baseline data and linked mortality records (389,843–452,189 participants), and electronic health records (EHR) from 45,515 UKB and 35,562 AoU participants. Multivariable regression and Cox models assessed lipid biomarkers, all‐cause mortality, cardiovascular mortality, and major adverse cardiovascular events (MACE). In UKB, *ε3ε4* (HR: 1.08, 95% CI: 1.01–1.15) and *ε4ε4* (HR: 1.54, 95% CI: 1.33–1.78) carriers had higher all‐cause mortality risk. In AoU, only *ε4ε4* carriers showed increased risk (HR: 1.64, 95% CI: 1.08–2.49). Cardiovascular mortality was assessed only in UKB, where *ε4ε4* carriers had an increased risk (HR: 1.30, 95% CI: 1.01–1.68). Mortality associations in UKB EHR data were consistent with those from baseline data and linked mortality records (e.g., *ε4ε4* genotype: all‐cause mortality HR: 1.51, 95% CI: 1.41–1.62; cardiovascular mortality HR: 1.54, 95% CI: 1.33–1.77). However, the statin:*APOE* interaction term included in the baseline analysis was not statistically significant. In AoU, changes in HDLC, LDLC, and triglycerides were associated with reduced all‐cause mortality risk. No significant MACE associations were observed in either cohort. This study reaffirms that *APOE ε4* genotype increases mortality risk, including in statin‐treated patients, and could therefore be used to inform enhanced monitoring or medication review in these patients.

## Graphical abstract

This study examined mortality in statin users from the UK Biobank and All of Us cohorts. Statins reduced deaths by 8%, while APOE *ε4ε4* carriers had 51%–64% higher mortality. In All of Us, higher HDL was linked to 74% fewer deaths, and lower LDL to 18% fewer. Findings may support enhanced monitoring or medication review in statin‐treated patients.

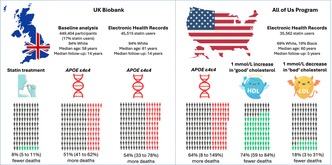

**Summary**

- What is the current knowledge on the topic?
  ○ *APOE* genotype may influence statin response.
- What question did this study address?
  ○ Our study examined the relationship between *APOE* genotype, statin use, and lipid level changes, all‐cause mortality, cardiovascular mortality, and major adverse cardiovascular events.
- What does this study add to our knowledge?
  ○ By analyzing UK Biobank and All of Us data, we found that statin treatment significantly altered HDL cholesterol and triglyceride levels.
  ○ In All of Us, changes in lipid levels (increased HDL cholesterol, decreased LDL cholesterol, and reduced triglycerides) were associated with lower all‐cause mortality.
  ○ *APOE* genotype significantly increased all‐cause mortality risk across both cohorts, especially among *ε4ε4* carriers.
- How might this change clinical pharmacology or translational science?
  ○ These results emphasize the association between *APOE* genotype and increased mortality risk, including in statin‐treated patients.
  ○ *APOE* genotype, when available, should inform enhanced monitoring and optimization of anti‐lipid therapies in these patients.

## 1 Introduction

Cardiovascular diseases (CVD) continue to be the leading cause of death globally, with ischemic heart disease accounting for the highest number of age‐standardized deaths in 2021, despite the COVID‐19 pandemic [1]. Statins (HMG‐CoA reductase inhibitors) have been shown to reduce CVD‐related mortality and are widely used for both primary and secondary prevention [2]. Apolipoprotein E (*APOE*) genotype has been reported to influence both cardiovascular risk and response to statin treatment [3, 4, 5, 6], although some studies have reported inconsistent findings [7]. Mutations in two SNPs (rs429358 and rs7412) create three main isoforms: *ε2* (mainly protective), *ε3* (wild type), and *ε4* (risk associated). The *ε2* isoform reduces cholesterol levels through lower low‐density lipoprotein (LDL) receptor binding, slower lipid clearance, and increased HMG‐CoA and LDL receptor synthesis, while *ε4* has the opposite effects [8, 9, 10]. However, carrying two *ε2* copies may impair lipid metabolism (familial dysbetalipoproteinemia), raising CVD risk [3, 11].

We recently conducted a meta‐analysis of 52 studies on *APOE* carrier status and statin response [12]. LDL cholesterol (LDLC), the biomarker most relevant to statin efficacy [13, 14], showed greater reductions in *ε2* carriers compared to *ε3* carriers, while *ε4* carriers had a reduced statin response [12]. Despite the inclusion of a significant number of studies, we identified some limitations, including considerable heterogeneity (*I* ^2^ > 70%, likely due to variations in study design, participant characteristics, genotyping procedures, etc.), a limited number of studies focusing on clinical outcomes such as mortality, an inability to account for specific statin types and doses used in the included studies, a lack of standardized reporting for genotypic and phenotypic data, and the inclusion of studies of unknown quality.

To address some of the above limitations, specifically adjusting for statin dose and type, and analyzing clinical outcomes like major adverse cardiovascular events (MACE), we conducted additional analyses using large datasets with linked electronic health records (EHR). Using both the UK Biobank (UKB; where > 90% of participants are White) [15, 16] and the All of Us Research Program (which is more diverse with ~70% White) [17, 18], we aimed to investigate the relationship between *APOE* genotype, statin use, and outcomes such as lipid level changes, all‐cause mortality, cardiovascular mortality, and MACE.

## 2 Methods

This study's reporting adheres to the REporting of studies Conducted using Observational Routinely collected health Data (RECORD) [19], STrengthening the REporting of Genetic Association Studies (STREGA) [20], and STrengthening the Reporting Of Pharmacogenetic Studies (STROPS) [21] guidelines, all of which are extensions of the Strengthening the Reporting of Observational Studies in Epidemiology (STROBE) [22] statement for observational genetic studies using routinely collected health data (Table S1). This study meets all five of the CODE‐EHR minimum framework standards for the use of structured health care data in clinical research, with three out of five standards meeting preferred criteria (Table S2) [23].

### 2.1 Data Sources

We used two cohorts for our analyses. The first, the UKB, is a population‐based prospective cohort that recruited over half a million participants from 22 assessment centers across England, Scotland, and Wales between 2006 and 2010 [15, 16]. At baseline, the UKB collected extensive phenotypic, health, lifestyle, and genome‐wide genotypic information. Mortality records linked to all participants are available. Approximately 230,000 participants have linked health care records, including death and cancer registries, hospital inpatient and outpatient episodes, and primary care data. Ethical approval was received by the UKB (North‐West Multicentre Research Ethics Committee approval number: 11/NW/0382), and written informed consent was received from all participants. Our study was approved by the UKB (application number: 56653).

We also used data from the All of Us (AoU) Research Program, launched by the US National Institutes of Health in 2018 to enhance diversity in biomedical research [17]. To date, the program has enrolled over 700,000 participants, with 80% from historically underrepresented backgrounds. Its Dataset v7 repository includes EHR and genomic data, accessible through a cloud‐based Researcher Workbench. Access to genomic data requires controlled tier access, granted after completing registration, training, and a data‐use agreement [17, 18]. Before participant recruitment and enrollment could commence, the AoU Institutional Review Board approved the program's protocol and materials.

### 2.2 Study Participants

We included only participants with genomic data who met genotype quality control criteria: (a) consistent genetic and self‐identified sex, (b) no sex chromosome aneuploidy, (c) non‐outliers for heterozygosity or missing rate, and (d) unrelated to other participants, as previously described [24].

For EHR analyses, additional inclusion criteria were: (a) prescription of one of the following statins: atorvastatin, cerivastatin, fluvastatin, lovastatin, pitavastatin, pravastatin, rosuvastatin, or simvastatin (note: lovastatin and pitavastatin are not available in the UK, and cerivastatin was withdrawn shortly after approval) [25], (b) more than 1 year of EHR data, (c) no statin prescription within the first year of registration (UKB) or first interaction with the health care system (AoU, defined as the first record in any of these Observational Medical Outcomes Partnership [OMOP] tables: “Condition Occurrence,” “Device Exposure,” “Drug Exposure,” “Measurement,” “Observation,” “Observation Period,” “Procedure Occurrence,” or “Visit Occurrence”), ensuring inclusion of only incident users, (d) statin treatment for at least 3 months (84 days), and for lipid outcome analyses, (e) at least two biomarker measurements—one before starting statins and one at least 28 days after.

To identify statin users, in UKB, we used Resource 592 (“Clinical coding classification systems and maps,” https://biobank.ndph.ox.ac.uk/ukb/refer.cgi?id=592) to match statin generic names to British National Formulary brand names, which were then mapped to prescription records. In AoU, we used the Anatomical Therapeutic Chemical (ATC) Classification System code “C10AA” (HMG‐CoA reductase inhibitors) along with curated phenotyping codes from the “All by All—Drug Phenotypes Curation” Workbench (https://workbench.researchallofus.org/workspaces/aou‐rw‐046fb18c/allbyalldrugphenotypescuration/data).

### 2.3 Outcome and Follow‐Up

We analyzed seven lipid biomarkers measured at baseline, detailed in the UKB category 17518 (https://biobank.ctsu.ox.ac.uk/ukb/label.cgi?id=17518): apolipoprotein A (Apo A, g/L), apolipoprotein B (Apo B, g/L), HDL cholesterol (HDLC, mmol/L), LDL directly measured cholesterol (LDLC, mmol/L), lipoprotein A (Lipo(a), nmol/L), total cholesterol (TC, mmol/L), and triglycerides (TG, mmol/L).

For EHR lipid biomarker analysis, we included those available for at least 781 eligible participants (see Section 2.5): four biomarkers from UKB (all in mmol/L: HDLC, LDLC, TC, and TG) and five from AoU (all in mg/dL: HDLC, non‐HDLC, LDLC, TC, and TG). For consistency, the AoU units (mg/dL) were converted to mmol/L using conversion factors of 88.57 for triglycerides and 38.67 for other lipid biomarkers. In the UKB, we identified biomarker measurements using Read v2 codes from the Health Data Research (HDR) UK Phenotype Library (https://phenotypes.healthdatagateway.org/phenotypes/) and a previous publication [26] (Table S3). To ensure data quality in the UKB, we excluded outlier values (< 0.1%), using thresholds defined by AoU clinical experts. For AoU, we used curated phenotyping codes from the “All by All—Lab Measurements Phenotypes Curation” Workbench (https://workbench.researchallofus.org/workspaces/aou‐rw‐0c74a4d9/allbyalllabmeasurementsphenotypescuration/data), harmonizing units, excluding outliers (boundaries set by clinical experts), and excluding data from sites with quality concerns post‐harmonization. In addition to analyzing net biomarker changes, we calculated percentage changes using the formula: percentage change = ([median biomarker value 4 weeks posttreatment − median biomarker value pretreatment]/median biomarker value pretreatment) × 100. Posttreatment values were taken as the median measurement starting 4 weeks after treatment initiation [12], allowing adequate time for statins to take effect. We tested various timeframes for capturing biomarker values: (a) 1 year before and no time limit after treatment (primary analysis with all values to maximize the sample size), (b) 1 year before and after treatment, (c) 6 months before and 1 year after treatment, and (d) 6 months before and after treatment.

The clinical outcomes assessed included all‐cause mortality, cardiovascular‐related deaths, and MACE. Cardiovascular deaths were identified using International Classification of Diseases (ICD)‐10 codes (I10–15, I44–51, I20–25, I61–73) following the European Systematic Coronary Risk Evaluation (SCORE) guidelines [27, 28]. MACE was defined per the Ramirez et al. [29] criteria, which included ICD‐9 and ICD‐10 codes for ischemic heart disease, myocardial infarction, heart failure, and ventricular arrhythmia. In the UKB, MACE cases were identified through Category 1712 (“Health‐related outcomes first occurrences,” https://biobank.ndph.ox.ac.uk/ukb/label.cgi?id=1712) from primary care data (Category 3000), hospital inpatient data (Category 2000), and death registry records (Fields 40001 and 40002). For AoU, MACE cases were identified from “Condition Occurrence,” “Observation,” “Measurement,” and “Procedure Occurrence” OMOP tables, with death data sourced from the “Death” tables. However, over 98% of deaths in AoU lacked cause information or matching concepts in the death table, preventing identification of cardiovascular‐related deaths in the AoU data.

Participants were followed from their baseline assessment until the occurrence of a clinical outcome or the censor date (31 December 2022). For EHR data, we assumed continuous statin exposure from the first statin prescription to the end of prescription records. Participants were followed from their first statin prescription until the earliest of the outcome occurrence (death or MACE) or the censor date (UKB: 31 December 2022 for mortality outcomes and 31 May 2016 for MACE [based on the earliest primary care censoring date, https://biobank.ndph.ox.ac.uk/ukb/exinfo.cgi?src=Data_providers_and_dates]; AoU: 1 July 2022).

### 2.4 Predictors

The exposure variable was *APOE* genotype (*ε2ε2*, *ε2ε3*, *ε2ε4*, *ε3ε3*, *ε3ε4*, and *ε4ε4*, determined based on the SNPs rs429358 and rs7412) [30] and statin use. Genotyping, imputation, and quality control procedures for both UKB and AoU have been described previously [15, 18].

The covariates adjusted for in the baseline analyses included age (in years) at recruitment, sex, body mass index (BMI, kg/m^2^), smoking status, alcohol consumption, race, level of physical activity, the genotyping array used, the Townsend index (a measure of socioeconomic status), and the first 10 principal components representing genetic ancestry (for measurement and coding details, see our previous paper) [24]. Comorbidities were also adjusted for including diabetes mellitus (based on self‐reported diabetes, doctor‐diagnosed diabetes, or use of antidiabetic drugs), hypertension (based on systolic and diastolic blood pressures, and the use of antihypertensives [renin‐angiotensin‐aldosterone system inhibitors, beta‐blockers, calcium channel blockers, and diuretics]), and CVD (based on self‐reported atrial fibrillation, coronary heart disease, stroke/transient ischemic attack, peripheral vascular disease, and heart failure or doctor‐diagnosed heart attack, angina, or stroke) [27, 31].

However, some of these covariates, for example, body mass index and alcohol/smoking habits, are not stable over time and may differ between the date of UKB recruitment and the date of the first statin prescription. Indeed, only 15.6%, 28.9%, and 66.0% of analyzed UKB participants had measurements for these covariates within 1, 2, and 5 years of the index date (start of follow‐up for the EHR analyses), respectively (see Figure S1). Given the potential changes by the index date, we limited EHR analyses to more stable factors: age at follow‐up start, sex, race, genotyping array (UKB only), the first 10 principal components of genetic ancestry, and comorbidities, including diabetes (ICD‐10: E10–E14), hypertension (ICD‐10: I10–I15), and CVD (ICD‐10: I20–25, I60–64, per American College of Cardiology/American Heart Association guidelines [27, 32], and MACE‐related codes [29]). We also included data provider (UKB only: Vision England, TPP England, Vision/EMIS Health Scotland, Vision/EMIS Health Wales, as AoU had over 45 sites for eligible participants), statin type, and statin strength as covariates. Lastly, net changes in lipid biomarkers were added as covariates to evaluate their association with all‐cause mortality. For HDLC, net changes were calculated as posttreatment minus pretreatment levels, with HRs reflecting the risk associated with a 1 mmol/L increase (HR < 1 indicates a beneficial effect). For other biomarkers, net changes were calculated as pretreatment minus posttreatment levels, with HRs representing the risk associated with a 1 mmol/L reduction (HR < 1 indicates a beneficial effect).

### 2.5 Sample Size and Power Analysis

The UKB baseline analyses included 389,843–452,189 participants, while EHR analyses included up to 45,515 UKB and 35,562 AoU participants. Sample size and power analyses are detailed in Data S1 and Figures S2 and S3.

### 2.6 Missing Data

Participants with missing outcome data were excluded from the corresponding analyses. The UKB datasets had < 1% participants missing data on at least one of the analyzed predictors, and these were excluded from the corresponding analyses. On the other hand, the AoU dataset had missing data on the strength of each statin tablet (e.g., 10 or 20 mg, etc.) for 14.7% of participants. Assuming statin strength was missing at random, we imputed these missing values using Multivariate Imputation by Chained Equations (MICE) in R [33] (Figure S4). The imputation model included all covariates (see Section 2.4) and outcomes (mortality, mace, pretreatment biomarker levels, and posttreatment biomarker levels) [34], with predictive mean matching as the imputation method. We generated 20 imputed datasets, exceeding the percentage of incomplete cases [35], and pooled estimates from the imputed datasets using Rubin's rules [36].

### 2.7 Statistical Analysis

For lipid outcomes (net or percentage changes in biomarker levels), we used linear regression models, and for mortality/MACE outcomes, we applied Cox proportional hazard models with competing risks (two terminal states) [37], such as all‐cause death for cardiovascular death/MACE, accounted for. When participants were diagnosed with MACE on the same day as their recorded death, they were classified as MACE cases, as death cannot occur before a MACE event. Each model adjusted for the covariates listed in Section 2.4, with certain categorical variables combined when the sample sizes were low. For example, statin types were grouped into three categories for the EHR analyses: simvastatin, atorvastatin, and other. For the UKB baseline analyses, a Statin:*APOE* interaction term was included. Effects were reported as regression coefficients for linear regression and hazard ratios (HRs) with 95% confidence intervals (CIs) for Cox regression. To assess the proportional hazards assumption for the Cox regression models, we used the coxph function from the R package survival [38]. To generate trend *p* values for *APOE* genotype, we used Type III ANOVA tests from the car package [39]. Statistical significance was set at *p* = 0.05, with Bonferroni correction (per outcome) for multiple testing.

For the clinical outcomes, we conducted a sensitivity analysis excluding individuals with dysbetalipoproteinemia, defined using previously reported screening criteria, as *ε2ε2* homozygotes with TC levels ≥ 200 mg/dL (5.2 mmol/L) and triglycerides ≥ 175 mg/dL (2.0 mmol/L) [40, 41]. Excluding these individuals was preferred over adjusting for dysbetalipoproteinemia to avoid multicollinearity, as all individuals with the condition had the *APOE ε2ε2* genotype. In a sensitivity analysis in the AoU dataset, we also analyzed only the participants with complete data. All analyses were conducted in R (version 4.4.1) [42].

### 2.8 Patient and Public Involvement

Patients and their representatives were not involved in formulating the research question or selecting outcomes for this analysis. However, they are actively engaged in both the UKB and the AoU Research Program.

## 3 Results

### 3.1 Participants

**FIGURE 1:**
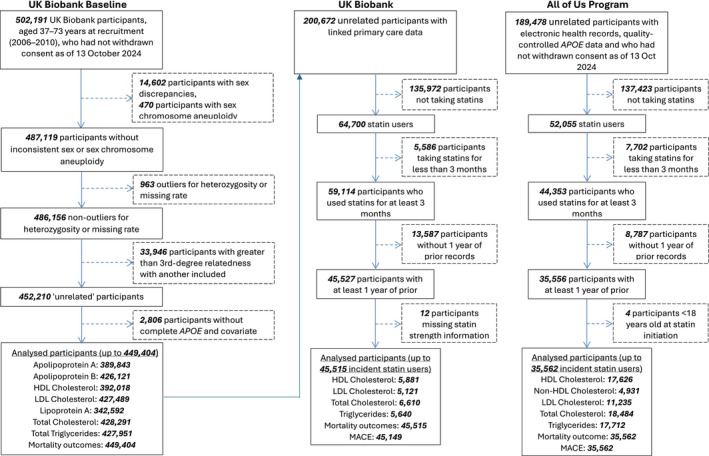
Flowchart for included participants. Bold values represent the total number of participants at each stage. *APOE*, apolipoprotein E; HDL, high‐density lipoprotein; LDL, low‐density lipoprotein; MACE, major adverse cardiovascular events.

As of 13 October 2024, a total of 502,191 UKB participants had not withdrawn consent. After applying exclusion criteria, the final analyzed cohort (baseline analysis) consisted of 449,404 participants (Figure 1). This cohort included 389,843 for ApoA, 426,121 for ApoB, 392,018 for HDLC, 427,489 for LDLC, 342,592 for Lipo(a), 428,291 for TC, 427,951 for TG, and 449,404 for mortality outcomes. From the UKB cohort used for baseline analysis, we selected 200,672 participants with linked primary care records (Figure 1). After exclusions based on statin use and registration record length, our analysis cohort for mortality outcomes included 45,515 incident statin users. Biomarker analyses were conducted on specific subsets: HDLC (5881), LDLC (5121), TC (6610), and TG (5640). MACE analyses included 45,149 participants. Using similar eligibility criteria in the AoU cohort, from 189,478 unrelated participants with EHR and quality‐controlled *APOE* data, we identified 35,562 incident statin users for mortality and MACE outcomes, with biomarker analyses on subsets: HDLC (17,626), non‐HDLC (4931), LDLC (11,235), TC (18,484), and TG (17,712).

Table 1 presents key characteristics of the analyzed participants, whereas Tables S4 and S5 provide additional details, including stratifications by mortality and biomarkers, as well as information on genetic ancestry principal components. For example, the median percentage changes in lipid biomarkers were: HDLC (0.0%), LDLC (−35.4%), TC (−22.4%), and TG (−13.1%) in the UKB cohort, and HDLC (2.3%), non‐HDLC (−26.2%), LDLC (−26.6%), TC (−16.6%), and TG (−8.16%) in the AoU cohort. Correlation analysis showed expected associations between the lipid markers (Figures S5 and S6).

**TABLE 1:** Characteristics of UK Biobank and All of Us program participants.

	UK Biobank baseline analysis (*N* = 449,404)	UK Biobank EHR (*N* = 45,515)	All of Us Program EHR (*N* = 35,562)
**Age (years)**			
Mean (SD)	56.6 (8.05)	60.3 (7.34)	59.1 (10.9)
Median [Min, Max]	58.0 [37.0, 73.0]	60.9 [26.7, 78.8]	59.8 [18.2, 98.0]
**Sex**			
Female	242,182 (53.9%)	9092 (41.9%)	19,123 (53.8%)
Male	207,222 (46.1%)	26,423 (58.1%)	16,439 (46.2%)
**Race**			
White	423,155 (94.2%)	42,915 (94.3%)	24,548 (69.0%)
Asian	10,204 (2.3%)	1496 (3.3%)	702 (2.0%)
Black	7134 (1.6%)	413 (0.9%)	6734 (18.9%)
Mixed/Other/Unknown	8911 (2.0%)	691 (1.5%)	3578 (10.1%)
**Hypertension history** ^a^			
No	348,628 (77.6%)	17,320 (38.1%)	10,660 (30.0%)
Yes	100,776 (22.4%)	28,195 (61.9%)	24,902 (70.0%)
**Diabetes mellitus history**			
No	425,577 (94.7%)	35,860 (78.8%)	24,245 (68.2%)
Yes	23,827 (5.3%)	9655 (21.2%)	11,317 (31.8%)
**Cardiovascular disease history**			
No	380,227 (84.6%)	27,377 (60.1%)	27,500 (77.3%)
Yes	69,177 (15.4%)	18,138 (39.9%)	8062 (22.7%)
**With dysbetalipoproteinemia**			
No	448,494 (99.8%)	45,353 (99.6%)	35,438 (99.7%)
Yes	910 (0.2%)	162 (0.4%)	124 (0.3%)
**Statin type**			
None	375,462 (83.5%)	0 (0.0%)	0 (0.0%)
Atorvastatin	16,621 (3.7%)	10,410 (22.9%)	17,883 (50.3%)
Cerivastatin	—	144 (0.3%)	9 (0.0%)
Fluvastatin	—	257 (0.6%)	1893 (5.3%)
Lovastatin	—	—	1019 (2.9%)
Pitavastatin	—	—	58 (0.2%)
Pravastatin	—	1014 (2.2%)	3127 (8.8%)
Rosuvastatin	—	583 (1.3%)	2913 (8.2%)
Simvastatin	51,863 (11.5%)	33,107 (72.7%)	8660 (24.4%)
Not specified	5458 (1.2%)	—	—
**Statin strength**			
Mean (SD)	—	28.9 (14.4)	28.1 (19.1)
Median [Min, Max]	—	40.0 [0.100, 80.0]	20.0 [0.300, 80.0]
Missing^b^		—	5222 (14.7%)
***APOE* genotype**			
*ε2ε2*	2828 (0.6%)	229 (0.5%)	231 (0.6%)
*ε2ε3*	54,901 (12.2%)	4436 (9.7%)	3691 (10.4%)
*ε2ε4*	11,271 (2.5%)	1019 (2.2%)	784 (2.2%)
*ε3ε3*	263,996 (58.7%)	26,963 (59.2%)	21,702 (61.0%)
*ε3ε4*	105,722 (23.5%)	11,646 (25.6%)	8266 (23.2%)
*ε4ε4*	10,686 (2.4%)	1222 (2.7%)	888 (2.5%)
**All‐cause death**			
Censored	410,105 (91.3%)	40,003 (87.9%)	34,842 (98.0%)
Got event	39,299 (8.7%)	5512 (12.1%)	720 (2.0%)
**Cardiovascular‐related death**			
Censored	437,401 (97.3%)	43,283 (95.1%)	—
Got event	12,003 (2.7%)	2232 (4.9%)	
**Death follow‐up time (years)**			
Mean (SD)	13.4 (2.05)	13.6 (4.76)	6.80 (5.53)
Median [Min, Max]	13.8 [0.0110, 16.8]	13.5 [0.282, 33.8]	5.43 [0.230, 34.2]
**MACE**			
Censored	—	33,510 (73.6%)	25,481 (71.7%)
Got event	—	11,639 (25.6%)	10,081 (28.3%)
Missing		366 (0.8%)	—
**MACE follow‐up time (years)**			
Mean (SD)	—	5.94 (4.44)	5.13 (4.99)
Median [Min, Max]	—	5.29 [0.00274, 26.5]	3.54 [0.00274, 32.4]
Missing		366 (0.8%)	—

Abbreviations: *APOE*, apolipoprotein E; BiLEVE, Biobank lung exome variant evaluation; EHR, electronic health records; MACE, major adverse cardiovascular events; Max, maximum; Min, minimum; *N*, sample size; SD, standard deviation.

^a^ This is “On antihypertensive medication” for the UK Biobank baseline analysis.

^b^ 12 (0.02%) UK Biobank participants missing strength information not included.

### 3.2 Lipid Outcomes

#### 3.2.1 UKB Baseline Analysis

Table S6 summarizes associations between covariates and lipid biomarkers in the UKB baseline analysis. Statin use and *APOE* genotype were significantly associated with all lipid biomarkers (statin use: *p* ≤ 5.97e‐10; APOE genotype: *p* < 2.00e‐16). Except for Lipo(a) (*p* = 0.104), all biomarkers met the Bonferroni‐adjusted significance threshold (*p* < 0.007) for APOE:statin interactions.

**FIGURE 2:**
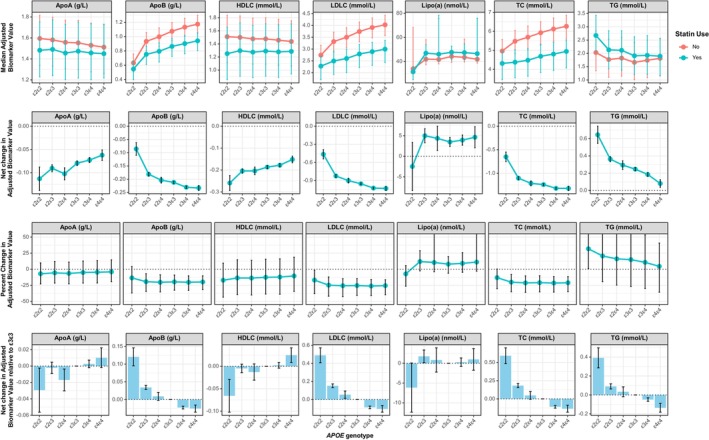
Associations between *APOE* genotype, statin use, and lipid biomarkers in the UK Biobank baseline analysis. This figure shows the median adjusted lipid biomarkers (top row), adjusted net changes (second row), adjusted percent changes (third row), and adjusted net change relative to the *ε3ε3* genotype (bottom row) across different *APOE* genotypes. The lipid biomarkers analyzed include apolipoprotein A (ApoA), apolipoprotein B (ApoB), HDL cholesterol (HDLC), LDL cholesterol (LDLC), lipoprotein A (Lipo(a)), total cholesterol (TC), and triglycerides (TG). In the bottom row, for ApoA and HDLC (increase beneficial), positive values indicate more benefit with statins relative to the *ε3ε3* genotype, while for other biomarkers (reduction beneficial), negative values indicate more benefit. Units for each biomarker are specified in their respective plots, with error bars representing 95% confidence intervals. Adjusted biomarker values are based on predictions from models adjusted for age at recruitment, sex, body mass index, smoking status, alcohol consumption, race, physical activity level, genotyping array, Townsend index, the first 10 principal components of genetic ancestry, and the interaction between *APOE* genotype and statin use.

Figure 2 illustrates these interactions, showing adjusted median biomarker values, net and percent changes, and differences relative to the *ε3ε3* genotype. Statin users had more favorable lipid profiles, with reduced LDLC and TC levels. Compared to *ε3ε3*, *ε4* carriers showed greater reductions in ApoB, LDLC, TC, and TG, alongside larger increases in ApoA and HDLC, suggesting larger statin benefits. In contrast, *ε2* carriers showed the opposite effects.

#### 3.2.2 EHR Analyses

Tables S7 (net change) and S8 (percentage change) summarize associations between covariates and lipid biomarkers, whereas Figure S7 shows *APOE* genotype effects on these changes. After Bonferroni correction (UKB: *p* < 0.0125; AoU: *p* < 0.01), only net changes in HDLC (AoU, *p* < 0.001) and TG (*p* < 0.001 in both cohorts) were significant, with HDLC in UKB (*p* = 0.024) slightly above the threshold. Percentage changes, accounting for baseline differences, showed significant results for HDLC (*p* = 0.003 in both cohorts) and TG (*p* < 0.001 in both cohorts). Excluding individuals with dysbetalipoproteinemia (0.3%–0.4%) produced similar results (Figure S8), though *ε2ε2* homozygotes aligned better with other *ε2* carriers, albeit with wider confidence intervals due to smaller sample sizes.

Initial analyses maximized sample size by omitting time limits, but additional analyses with time restrictions (Figure S9) reduced sample sizes: 60%–75% of the original sample size for 1 year pre/post statin initiation, 50%–60% for 6 months pre/1 year post, and 30%–45% for 6 months pre/post. Figure S10 shows percentage changes in lipid biomarkers by *APOE* genotype across these time windows, with confidence intervals widening as sample sizes decreased.

### 3.3 Mortality and MACE Outcomes

#### 3.3.1 UKB Baseline Analysis

**FIGURE 3:**
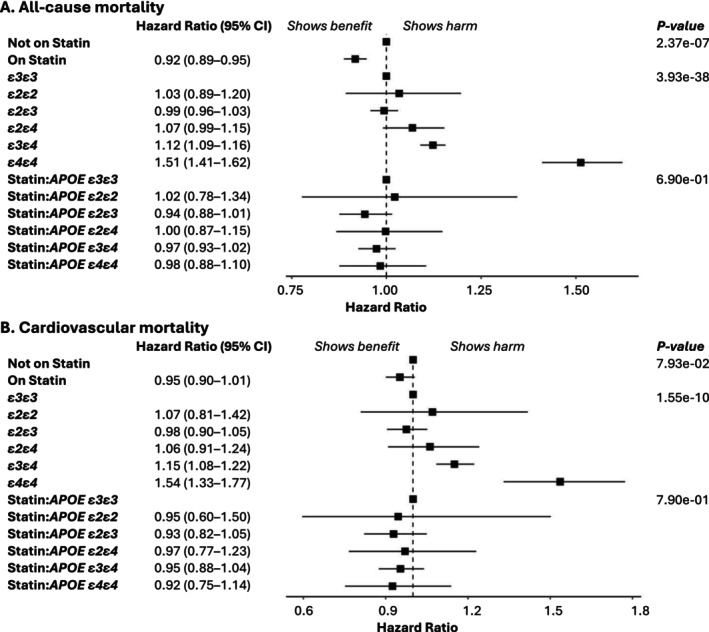
Associations between *APOE* genotype, statin use, and mortality outcomes in the UK Biobank baseline analysis. This figure presents hazard ratios and 95% confidence intervals (CIs) for (A) all‐cause death and (B) cardiovascular death, stratified by statin use, apolipoprotein E (*APOE*) genotype, and the statin:*APOE* interaction. Genotype effect estimates reflect associations across the entire cohort, including both statin users and nonusers. The analysis adjusts for multiple covariates, including age, sex, body mass index, race, smoking status, alcohol consumption, physical activity, Townsend deprivation index, systolic and diastolic blood pressure, use of antihypertensive medications, diabetes mellitus history, cardiovascular disease history, genotyping array, and the first 10 principal components of genetic ancestry.

Table S9 shows associations between covariates and mortality outcomes, whereas Figure 3 highlights the effects of *APOE* genotype and statin use. In Panel A, statin use significantly reduced all‐cause mortality risk (HR: 0.92, 95% CI: 0.89–0.95, *p* = 2.37e‐07). *APOE* stratification showed increased risk for *ε4* carriers, particularly *ε4ε4* (HR: 1.51, 95% CI: 1.41–1.62, *p* < 2.00e‐16), while *ε2* carriers had neutral or slightly lower risk. The statin:*APOE* interaction was not significant. Sensitivity analyses (Table S9) excluding 910 individuals with dysbetalipoproteinemia (0.2% of the sample) showed consistent results. A proportional hazards assumption test (Figure S11) indicated time‐dependent effects, leading to a sensitivity analysis stratifying follow‐up at 7 and 12 years. Results (Table S10) were consistent, with statin HRs of 0.90, 0.92, and 0.93 across time periods, averaging the 0.92 seen in Figure 3.

Panel B of Figure 3 shows hazard ratios for cardiovascular mortality. Statin use had a protective but nonsignificant effect (HR: 0.95, 95% CI: 0.90–1.01, *p* = 0.079). *ε4* carriers, especially *ε4ε4*, had a higher cardiovascular mortality risk (HR: 1.54, 95% CI: 1.33–1.77, *p* = 4.83e‐09). As with all‐cause mortality, the statin:*APOE* interaction was not significant.

#### 3.3.2 EHR Analyses

**FIGURE 4:**
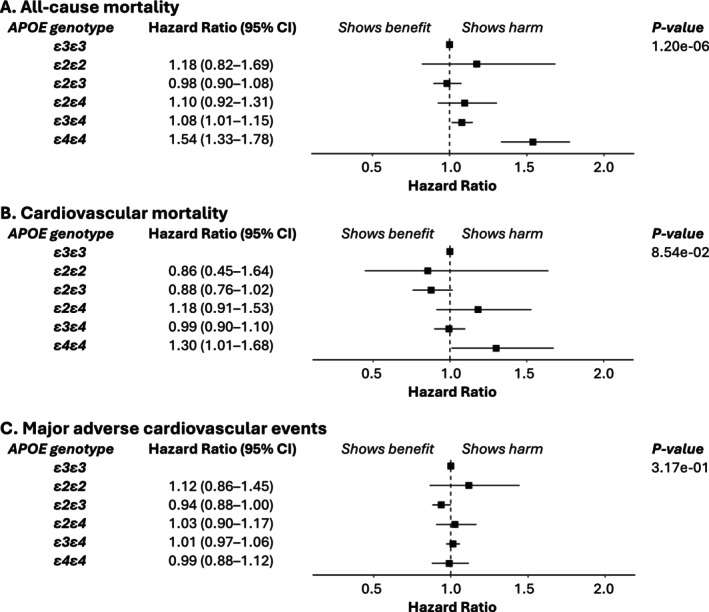
Associations between *APOE* genotype and clinical outcomes in the UK Biobank Electronic Health Records (statin‐treated patients). This figure shows hazard ratios and 95% confidence intervals for three clinical outcomes: (A) All‐cause death, (B) Cardiovascular death, and (C) Major adverse cardiovascular events. The analyses are adjusted for the following covariates: age, sex, histories of hypertension, diabetes mellitus, and cardiovascular disease, data provider, type and strength of statin, genotyping array, and the first 10 principal components of genetic ancestry. *APOE*, apolipoprotein E.

**FIGURE 5:**
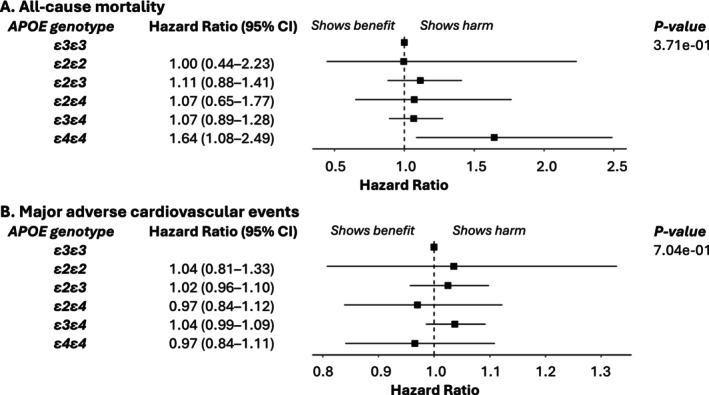
Associations between *APOE* genotype and clinical outcomes in the All of US Program (statin‐treated patients). This figure shows hazard ratios and 95% confidence intervals for three clinical outcomes: (A) All‐cause death and (B) Major adverse cardiovascular events. The analyses are adjusted for the following covariates: age, sex, histories of hypertension, diabetes mellitus, and cardiovascular disease, type and strength of statin, and the first 10 principal components of genetic ancestry. *APOE*, apolipoprotein E.

Table S11 summarizes associations between covariates and mortality, whereas Figures 4 (UKB) and 5 (AoU) highlight the effects of *APOE* genotype in statin‐treated patients. In UKB (Figure 4, Panel A), *ε4ε4* carriers had a significantly higher all‐cause mortality risk (HR: 1.54, 95% CI: 1.33–1.78), while *ε3ε4* carriers had a modest increase (HR: 1.08, 95% CI: 1.01–1.15). In AoU (Figure 5, Panel A), *ε4ε4* carriers showed a significant risk increase (HR: 1.64, 95% CI: 1.08–2.49), whereas *ε3ε4* carriers did not (HR: 1.07, 95% CI: 0.89–1.28). Sensitivity analyses, including dysbetalipoproteinemia exclusions and complete‐case analysis, produced similar findings (Table S11). Cardiovascular mortality (UKB only, Figure 4, Panel B) was significantly elevated in *ε4ε4* carriers (HR: 1.30, 95% CI: 1.01–1.68). MACE analysis (Table S12) found no significant *APOE* associations in either cohort (Figures 4 and 5, Panel C).

**FIGURE 6:**
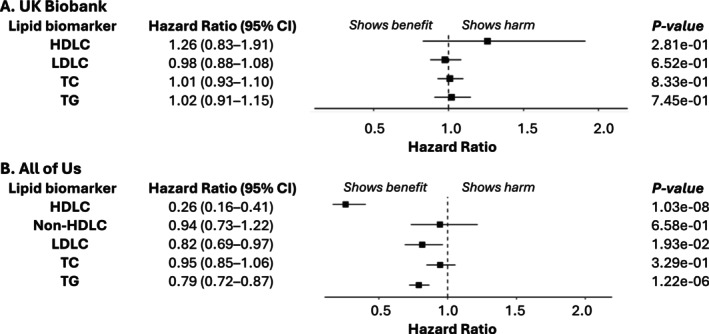
Associations between net changes (mmol/L) in lipid biomarkers and all‐cause mortality in statin‐treated patients. Panels A and B show hazard ratios (HRs) with 95% confidence intervals (CIs) for the UK Biobank and All of Us cohorts, respectively. Net changes in HDLC were calculated as posttreatment minus pretreatment biomarker levels, meaning HRs represent the risk associated with a 1 mmol/L increase in HDLC (HR < 1 indicates a beneficial effect). For all other biomarkers, net changes were calculated as pretreatment minus posttreatment levels, so HRs reflect the risk associated with a 1 mmol/L reduction (HR < 1 indicates a beneficial effect). Analyses were adjusted for age, sex, histories of hypertension, diabetes mellitus, and cardiovascular disease, data provider (UK Biobank), type and strength of statin, genotyping array (UK Biobank), *APOE* genotype, and the first 10 principal components of genetic ancestry. *APOE*, apolipoprotein E; HDLC, high‐density lipoprotein cholesterol; LDLC, low‐density lipoprotein cholesterol; TC, total cholesterol; TG, triglycerides.

Figure 6 examines lipid biomarker changes and all‐cause mortality. Median net changes (mmol/L) were: UKB, HDLC: 0.00, LDLC: −1.25, TC: −1.30, TG: −0.20; AoU, HDLC: 0.03, non‐HDL: −0.98, LDLC: −0.82, TC: −0.85, TG: −0.10 (Table S4). In UKB, changes were not significantly associated with mortality. In AoU, increased HDLC was strongly protective (HR: 0.26, 95% CI: 0.16–0.41 per mmol/L), while reductions in LDLC (HR: 0.82, 95% CI: 0.69–0.97) and TG (HR: 0.79, 95% CI: 0.72–0.87) were linked to lower mortality risk.

## 4 Discussion

Using UKB and AoU data, we examined *APOE* genotype, statin use, and various clinical outcomes. As would be expected, each 1 mmol/L change in lipids associated with statin use significantly affected mortality risk (see discussion of these known associations in Data S2). *APOE* genotype significantly affected net and percentage changes only for HDLC and triglycerides, while LDLC, the most mortality‐relevant biomarker, showed no significant change. However, *ε2ε3* carriers had greater LDLC reductions than *ε3ε3*, while *ε3ε4* and *ε4ε4* had smaller reductions (Figure S7) aligning with prior findings that *ε2* carriers respond better to statins, while *ε4* carriers are less responsive [8, 9, 10]. By contrast, *ε4* carriers had greater LDLC reductions in the baseline analysis (Figure 2), but this may be related to the fact that non‐statin *ε4* carriers had much higher baseline LDLC levels than *ε2* carriers. This is consistent with the findings of Tavintharan et al. [43] who showed that higher baseline LDLC levels in *ε4* carriers result in greater percentage reductions with statin therapy compared to *ε2* or *ε3* carriers with lower baseline levels. However, despite this, statin‐treated *ε4ε4* individuals still had higher LDLC levels than untreated *ε2ε2* individuals, suggesting a degree of resistance to the effects of statins in *ε4* carriers. These findings also highlight the limitations of population‐level analysis, which do not apply to our individual‐level EHR analyses from UKB and AoU.

The lack of statistical significance for LDLC changes is probably due to the relatively small sample size and even smaller genotype subgroups. To address this, a post hoc analysis was performed by grouping *ε2ε2* (without dysbetalipoproteinemia) and *ε2ε3* carriers into an “*ε2* carrier” group and combining *ε3ε4* and *ε4ε4* carriers into an “*ε4* carrier” group. *ε2ε4* carriers were assigned to either the “*ε2* carrier” group (UKB) or the “*ε4* carrier” group (AoU), based on genotype‐specific effect directions. This grouping improved statistical power, decreasing the LDLC *p* value from 0.049 to 0.012 in the UKB cohort and from 0.223 to 0.030 in the AoU cohort. The percentage changes which reflect population averages are shown in Figure S7; the wide confidence intervals reflect not only the small sample size, but also the high variability within the population, suggesting that changes may be substantial for certain individuals, and therefore the importance of in‐depth clinical assessment at the individual level. In the post hoc analysis, *ε2ε4* carriers were classified under both *ε2* and *ε4* carriers, a common practice [12]. However, the literature lacks consensus on how to handle the *ε2ε4* genotype (whether to exclude it or classify it as *ε2*, *ε3*, or *ε4*) and grouping *ε2ε2* with *ε2ε3* may be misleading, given the former's association with an increased risk of familial dysbetalipoproteinemia [3, 11, 12]. These complexities informed our decision to focus on *APOE* genotypes rather than carrier status. Moreover, genotype‐level effect estimates can be pooled to analyze carrier‐level effects, but the reverse is not possible, highlighting the flexibility and interpretive advantages of genotype‐specific analyses.

To maximize sample size, we analyzed the association between *APOE* genotypes and clinical outcomes without adjusting for lipid biomarker changes, as these data were available for only a small fraction of participants (one‐seventh in UKB and half in AoU). Consistent with prior evidence linking the *ε4* allele to adverse cardiovascular and metabolic profiles [8, 9, 10], which may contribute to increased mortality, the *ε4* allele was significantly associated with all‐cause mortality in the UKB cohort (*N* = 45,515). Specifically, *ε3ε4* carriers had a modestly elevated risk (HR: 1.08, 95% CI: 1.01–1.15), while *ε4ε4* carriers showed a substantially higher risk (HR: 1.54, 95% CI: 1.33–1.78) of all‐cause mortality, which decreased to a HR of 1.30 (95% CI: 1.01–1.68) for cardiovascular mortality. These findings were consistent with the analyses of the UKB baseline assessment data and linked mortality records (452,189 participants), where *ε4ε4* carriers had a hazard ratio of 1.51 (95% CI: 1.41–1.62) for all‐cause mortality and 1.54 (95% CI: 1.33–1.77) for cardiovascular mortality. In the smaller AoU cohort (*N* = 35,562), only *ε4ε4* carriers similarly showed a significantly increased risk of all‐cause mortality (HR: 1.64, 95% CI: 1.08–2.49) with *ε3ε4* carriers showing a nonsignificant increase (HR: 1.07, 95% CI: 0.89–1.28). These findings are consistent with our power analyses, which indicated greater power to detect associations in the larger UKB cohort.

Despite several strengths, including two relatively large cohorts and robust associations supported by sensitivity analyses (e.g., excluding dysbetalipoproteinemia participants and conducting complete‐case analysis for AoU), our study had limitations. These included low statistical power for some outcomes and the inability to incorporate relevant covariates such as body mass index or adherence to statin treatment, either due to challenges in accurate phenotyping or the absence of these variables in the EHR used [44]. The lack of a genotype × treatment interaction also probably reflects a lack of statistical power. In the AoU cohort, approximately 15% of participants had missing statin strength data; however, these missing values were addressed using multiple imputation. Additionally, the representativeness of the cohorts should be considered; for example, UKB participants have been reported to be, on average, healthier than the general population [45]. Finally, the fasting status of EHR biomarker measurements included a mix of fasting, non‐fasting (random), and unknown states, which likely introduced variability. However, this approach is consistent with previous studies [26].

In conclusion, our analysis highlights the impact of *APOE* genotype on clinical outcomes, particularly all‐cause mortality. Consistent with prior evidence, statin‐induced increases in HDLC and reductions in LDLC and triglycerides were associated with lower all‐cause mortality. Larger studies are needed to further strengthen the evidence base and explore associations with other clinical outcomes that could not be detected due to limited statistical power. Our results emphasize the importance of exploring the interplay between *APOE* genotype, lipid profiles, and clinical outcomes, particularly in diverse populations and varying cohort contexts. They also reaffirm that *APOE ε4* genotype increases mortality risk, including in statin‐treated patients, and could therefore be used to inform enhanced monitoring or medication review in these patients.

## Author Contributions

All authors wrote the manuscript and designed the research. I.G.A. performed the research and analyzed the data.

## Conflicts of Interest

M.P. currently receives partnership funding, paid to the University of Liverpool, for the following: MRC Clinical Pharmacology Training Scheme (co‐funded by MRC and Roche, UCB, Eli Lilly, and Novartis), and the MRC Medicines Development Fellowship Scheme (co‐funded by MRC and GSK, AZ, Optum, and Hammersmith Medicines Research). He has developed an HLA genotyping panel with MC Diagnostics but does not benefit financially from this. He is part of the IMI Consortium ARDAT (www.ardat.org); none of these funding sources have been used for the current research. All other authors declared no competing interests for this work.

## Supporting information


**Tables S1–S12:** cts70314‐sup‐0001‐TablesS1‐S12.xlsx.

**Appendix S1:** cts70314‐sup‐0002‐AppendixS1.docx.

## Data Availability

The data that support the findings of this study are available from UK Biobank (https://www.ukbiobank.ac.uk/) and All of Us Research Program (https://allofus.nih.gov/), with the permission of UK Biobank and All of Us Research Program, respectively.

## Appendix 1 Multimorbidity Mechanism and Therapeutic Research Collaborative

Adam Butterworth, Alasdair Warwick, Alba Fernandez‐Sanles, Albert Henry, Alvina G. Lai, Amanda Roberts, Andrea Jorgensen, Ana Torralbo, Anoop D. Shah, Aroon Hingorani, Arturo Gonzalez‐Izquierdo, Maria Carolina Borges, Caroline Dale, Chris Finan, Claudia Langenberg, Daniel C. Alexander, Deborah Lawlor, Diana Dunca, Eda B. Ozyigit, Amand F. Schmidt, Harry Hemingway, Honghan Wu, Innocent G. Asiimwe, Jasmine Gratton, Jean Gallagher, Jorgen E. Engmann, Lauren E. Walker, Victoria L. Wright, Magdalena Zwierzyna, Margaret Ogden, Martin Cox, Mary Mancini, Michail Katsoulis, Mira Hidajat, Munir Pirmohamed, Natalie Fitzpatrick, Nishi Chaturvedi, Rashmi Kumar, Rohan Takhar, Sandesh Chopade, Simon Ball, Spiros Denaxas, Tina Shah, Valerie Kuan, Nikita Hukerikar, Reecha Sofat, Frances Bennett, David Ryan, Maik Pietzner.
