# Supplementary material for: APOE Genotype and Statin Response: Evidence From the UK Biobank and All of Us Program
Source: Clin Transl Sci. 2025 Aug 5;18(8):e70314. doi: 10.1111/cts.70314 (PMC12324813; doi:10.1111/cts.70314)
Supplement: Supplementary file 2 — Appendix S1: cts70314‐sup‐0002‐AppendixS1.docx. [file CTS-18-e70314-s002.docx]

***APOE* Genotype and Statin Response: Evidence from the UK Biobank and All of Us Program.**

Innocent G. Asiimwe, PhD^1,2^, Andrea L. Jorgensen, PhD^2^, Munir Pirmohamed, PhD^1^, on behalf of the Multimorbidity Mechanism and Therapeutic Research Collaborative (MMTRC).

^1^Department of Pharmacology and Therapeutics, Institute of Systems, Molecular and Integrative Biology, University of Liverpool, Liverpool, UK.

^2^Department of Health Data Science, Institute of Population Health Sciences, University of Liverpool, Liverpool, UK.

**Contents**

**Supplementary Text……………………………………………………………………………………………2**

**Supplementary Figures……………………………………………………………………………………….4**

**Supplementary References……………………………………………………………………………..…22**

**Supplementary Text**

**Data S1:** **Sample size and Power analysis**

***UK Biobank Baseline analysis***

Since we used an available cohort, we focused on calculating the available statistical power instead of determining the necessary sample size. For the lipid biomarkers, we used the power_envir.calc.linear_outcome function from the R package genpwr^1^ to calculate power, using the sample sizes and standard deviations of the seven outcomes available from UK Biobank participants (<https://biobank.ctsu.ox.ac.uk/ukb/label.cgi?id=17518>). Assuming a target coefficient of determination (*R²*) of 1% for genetic, environmental, and gene-environment interaction effects, an additive mode of inheritance, a minor allele frequency as low as 1%, and approximately 17% statin use,^2^ we found that any sample size above 10,000 would yield statistical power close to 100%. This was determined using a Bonferroni-corrected *P*-value for the seven biomarkers (*P* = 0.05/7), indicating that our study was well-powered for all lipid biomarker outcomes.

For the two clinical outcomes (all-cause mortality and cardiovascular-related mortality), we used the powerEpiInt function from the R package powerSurvEpi^3^ to calculate statistical power. This function requires a pilot dataset and binary versions of the interacting variables (statins and *APOE*). For each genotype contrast compared to the reference genotype (*ε3ε3*), we randomly selected 1,000 participants from the UK Biobank cohort. As shown in Figure S2, assuming a significance level of 0.05, a sample size of 449,404 was sufficient to detect effect sizes for all-cause mortality across all genotype comparisons at an 80% power threshold, with a minimum hazard ratio (HR) of 1.1 (an HR of at least 1.2 was required for the *ε2ε2* genotype). For cardiovascular-related deaths, higher HRs were necessary to achieve over 80% power: HR = 2.3 for *ε2ε2*, 1.3 for *ε2ε3*, 1.5 for *ε2ε4*, 1.2 for *ε3ε4*, and 1.5 for *ε4ε4*.

***Electronic Health Record Analyses***

We aimed to analyse all eligible participants in the available cohorts to maximize sample size, so we report statistical power rather than minimum sample size requirements for clinical outcomes. For lipid biomarkers, which had varying sample sizes, we calculated the minimum sample size needed for inclusion.

For lipid biomarker analysis (using percent change as the outcome), we determined the sample size with the ss.calc.linear function from the R package "genpwr".^1^ Assuming a target coefficient of determination (*R²*) of 1%, minor allele frequencies of 5–20%, 80% power, standard deviations of 10–30%, and an additive mode of inheritance, a minimum of 781 participants was required at *P* = 0.05. After applying Bonferroni correction for 2, 3, 4, and 5 biomarkers, the minimum sample sizes increased to 946, 1042, 1110, and 1163, respectively.

For clinical outcomes, we used the powerEpiInt function from the R package powerSurvEpi^3^ to estimate power, requiring a pilot dataset and two binary variables. We included sex (in addition to *APOE* genotype) due to its known influence on *APOE* effects in statin-treated patients.^4^ For each genotype contrast versus *ε3ε3*, we randomly selected 1,000 participants, with assumptions of a 0.05 significance level, effect sizes (HRs) of 1.1–2.3, and an 80% power threshold.

In the UKB, a sample size of 45,512 was adequate to detect all-cause mortality effects across genotypes with minimum detectable HRs ranging from 1.2 (*ε3ε4*) to 2.3 (*ε2ε2*) (Figure S2). For cardiovascular deaths, there was insufficient power for *ε2ε2*, with minimum HRs required for detection of other genotypes ranging from 1.3 to 1.8. For MACE (sample size: 45,146), *ε2ε2* was detectable at an HR of about 1.8, and other genotypes at HRs between 1.1 and 1.3. In AoU, a sample size of 35,562 was sufficient for detecting all-cause mortality effects only for the genotypes *ε2ε3* (HR = 2.3) and *ε3ε4* (HR > 1.5). For MACE, all genotypes were detectable, requiring HRs ranging from 1.1 to 1.8 (Figure S3).

**Data S2: Discussion of Known Findings**

Using UK Biobank (UKB) and All of Us (AoU) data, we examined *APOE* genotype, statin use, and various clinical outcomes. Consistent with existing evidence, increases in HDLC and reductions in LDLC and triglycerides were protective against all-cause mortality in the AoU cohort. In this cohort, the median net changes in lipid biomarkers (in mmol/L and percentage change) were as follows: HDLC: 0.03 (2%), LDLC: -0.82 (-27%), and triglycerides: -0.10 (-8.16%) while the UKB cohort changes were: HDLC: 0 (0%), LDLC: -1.25 (-35%), and triglycerides: -0.15 (-11.8%). These changes are consistent with the expected benefits of statins, which inhibit HMG-CoA reductase, the key enzyme regulating cholesterol synthesis. Statins typically lead to a 1–10% increase in HDLC, a reduction of approximately 30% in LDLC for low-intensity statins, a 30–49% reduction in LDLC for moderate-intensity statins, and a 10–20% reduction in triglycerides.^5, 6, 7, 8^ The UKB baseline cross-sectional analysis examined additional lipids and compared statin-treated and untreated individuals at a population level. Lipoprotein A was the only lipid unaffected by statin use, which is consistent with a previous systematic review.^9^ The cross-sectional analyses should be interpreted with caution, as they do not capture temporal changes. For example, in Figure 2 (top panel), it is unclear whether HDLC levels decreased as a result of statin use or if individuals prescribed statins already had low HDLC, which remained unchanged given statins' modest effect on HDLC. Similarly, we cannot determine whether triglyceride levels increased after statin use or if patients with already high triglycerides were more likely to be prescribed statins.

Each 1 mmol/L lipid change significantly affected mortality risk: HDLC (74% risk reduction, HR: 0.26, 95% CI: 0.16–0.41), LDLC (18%, HR: 0.82, 95% CI: 0.69–0.97), and triglycerides (21%, HR: 0.79, 95% CI: 0.72–0.87). Applying these estimates to median lipid changes in AoU, statins reduced all-cause mortality by ~2.2% (HDLC, 0.03 mmol/L increase × 74% reduction/mmol), 14.8% (LDLC), and 2.1% (triglycerides), with LDLC reduction providing the greatest benefit. This aligns with a meta-analysis of 90,056 participants across 14 randomized controlled trials (RCTs), which reported a rate ratio of 0.88 (95% CI: 0.84–0.91) per mmol/L LDLC reduction.^10^ The 1-year mean LDLC reduction in these RCTs (1.09 mmol/L) was comparable to our findings (AoU: -0.82, UKB: -1.25 mmol/L). While biomarker changes were observed in both cohorts, associations with all-cause mortality were significant only in AoU. This discrepancy may be attributed to sample size differences: AoU included >11,000 more participants in the HDLC analysis (17,626 vs. 5,736), >6,000 more participants in the LDLC analysis (11,235 vs. 5,052), and >12,000 more in the triglycerides analysis (17,875 vs. 10,977), potentially providing greater statistical power to detect significant associations.

**Supplementary Figures**

**
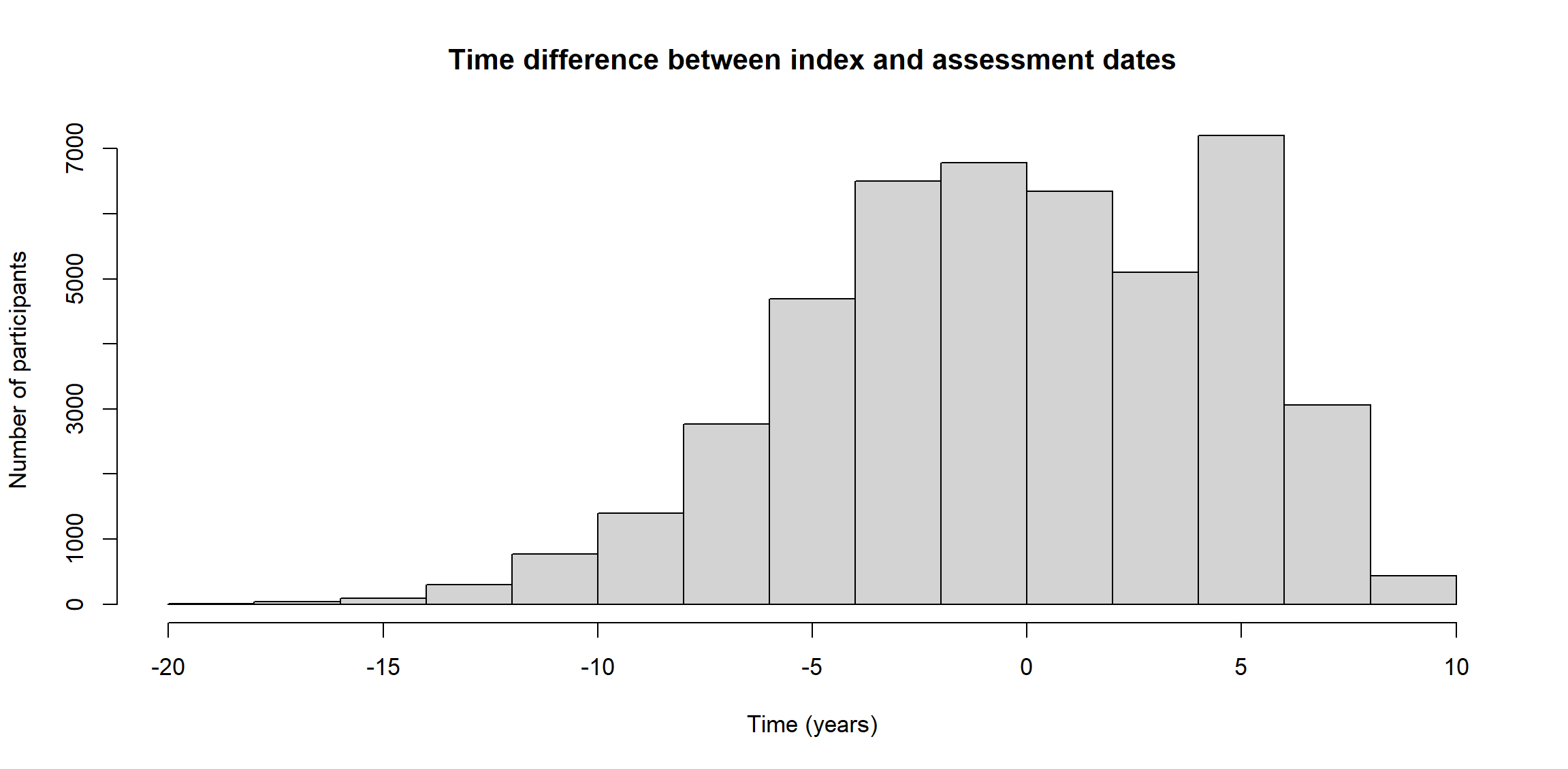
**

**Figure S1. Time difference between the index date (start of follow-up) and the date of attending a UK Biobank assessment centre.**

**
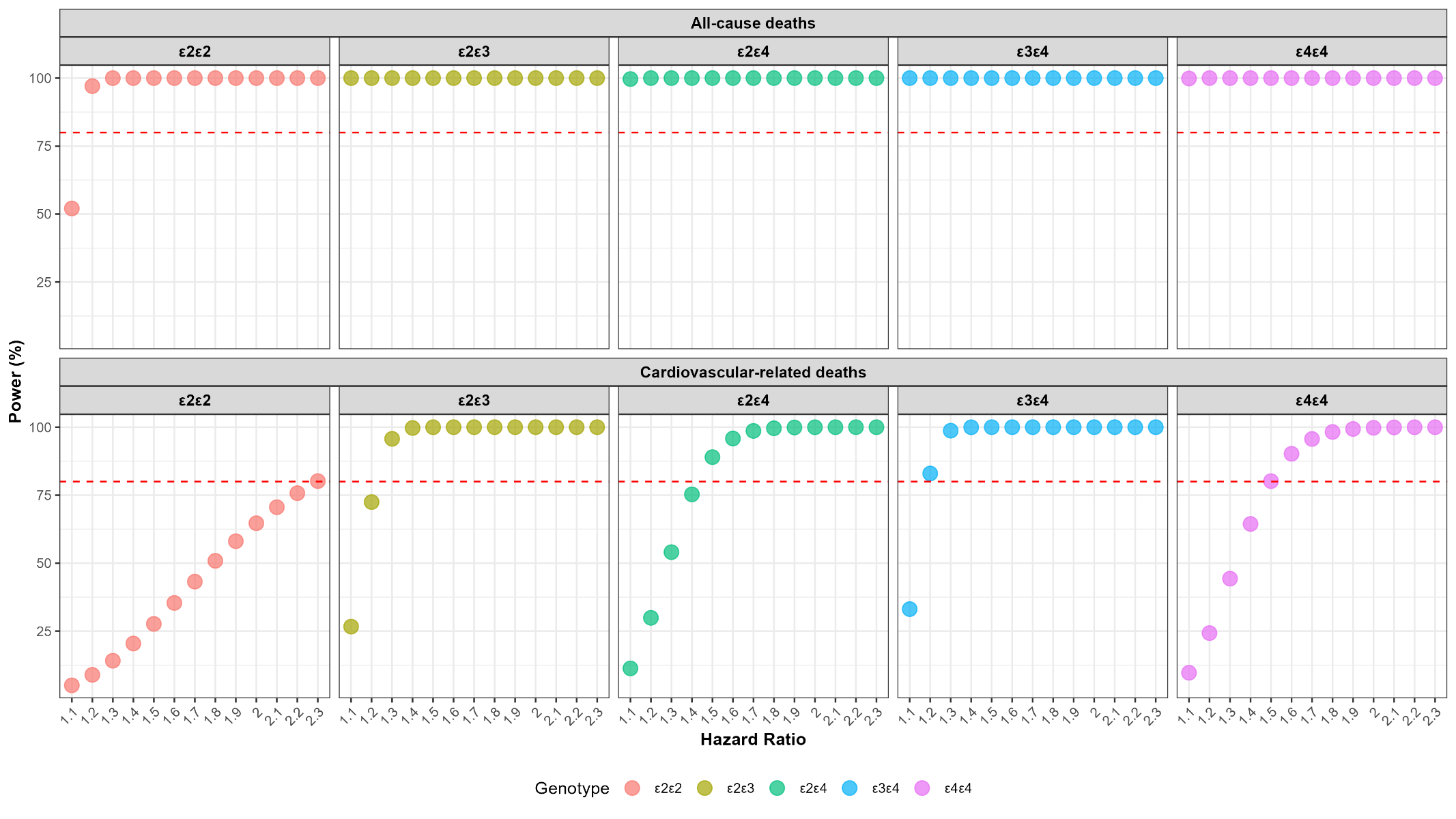
**

**Figure S2. Power analysis (UK Biobank baseline analysis) for varying hazard ratios for each *APOE* genotype, assuming a sample size of** **449,404, with *ε3ε3* as the reference genotype.** Panels display power (%) to detect hazard ratios for all-cause deaths (top) and cardiovascular-related deaths (bottom) across the genotypes *ε2ε2*, *ε2ε3*, *ε2ε4*, *ε3ε4*, and *ε4ε4*. The dashed red line indicates the 80% power threshold.

**A. UK Biobank**


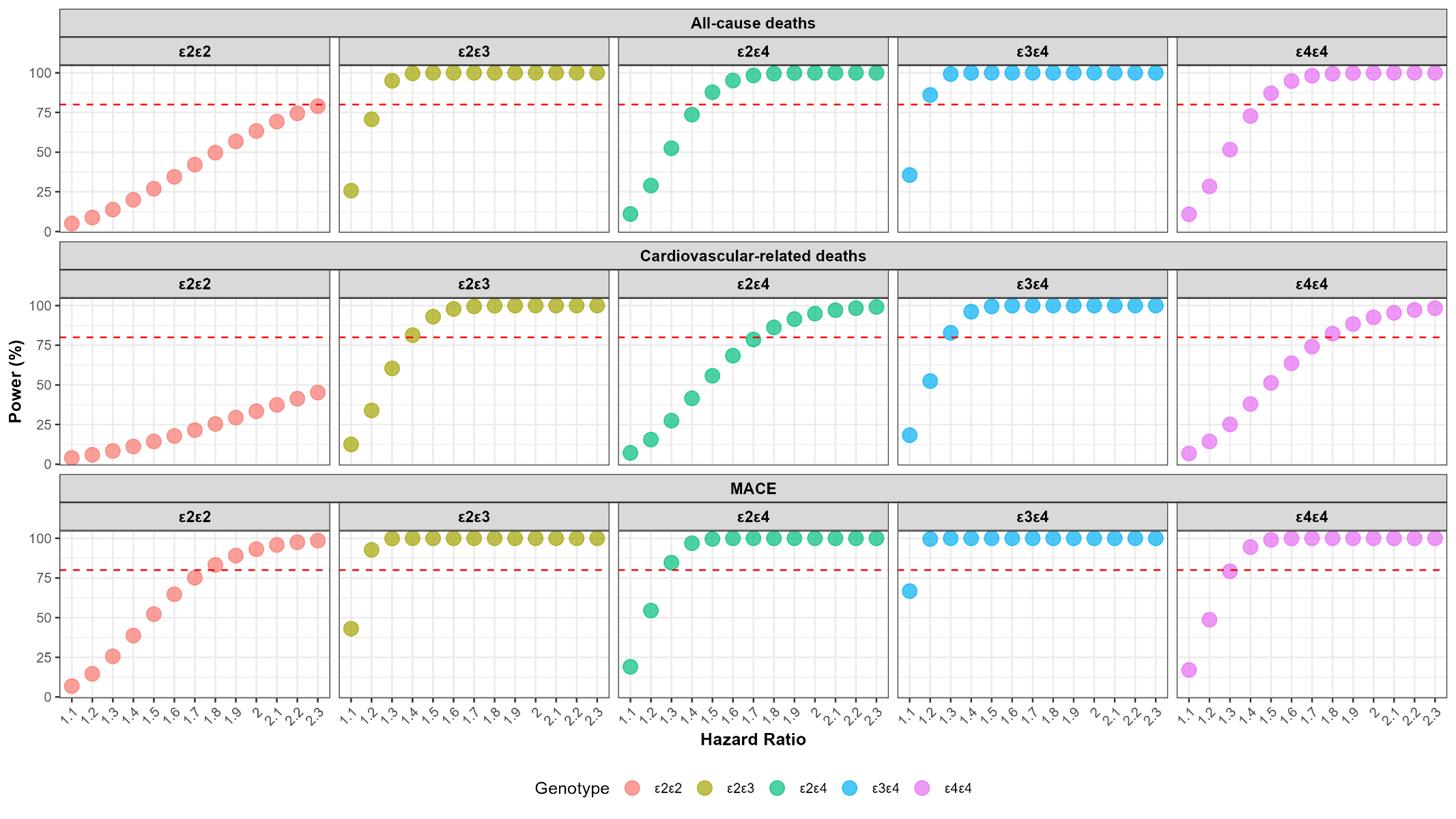


**B. All of Us**

**
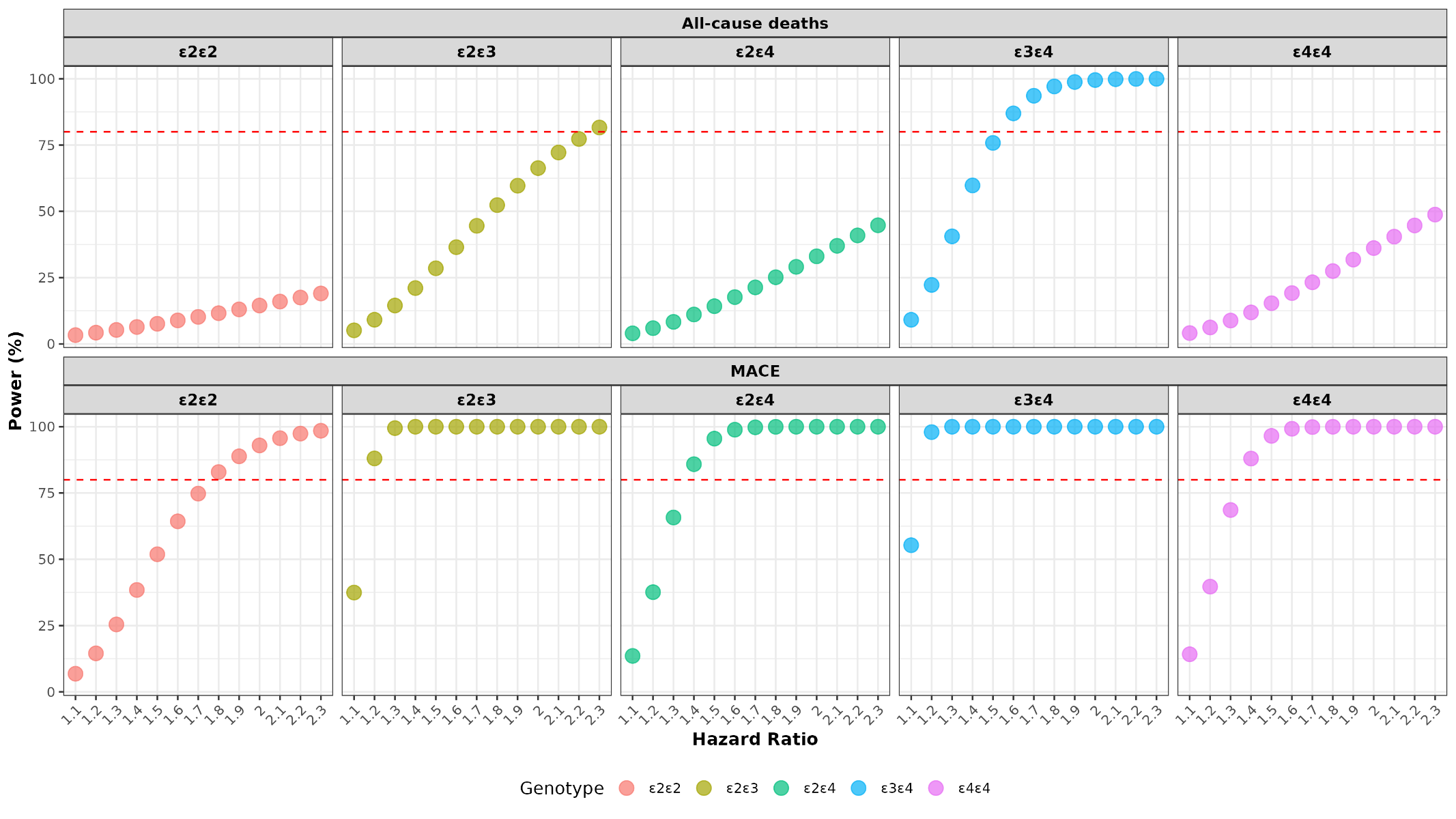
**

**Figure S3. Power analysis (electronic health records) for varying hazard ratios for each *APOE* genotype, with *ε3ε3* as the reference genotype. A. UK Biobank.** Sample sizes of 45,515 and 45,149 were assumed for mortality and major adverse cardiovascular events (MACE) outcomes, respectively. **B. All of Us Program.** A sample size of 35,562 was assumed. Panels display power (%) to detect hazard ratios across the genotypes *ε2ε2*, *ε2ε3*, *ε2ε4*, *ε3ε4*, and *ε4ε4*. The dashed red line indicates the 80% power threshold.

**A. Convergence Plot**

**
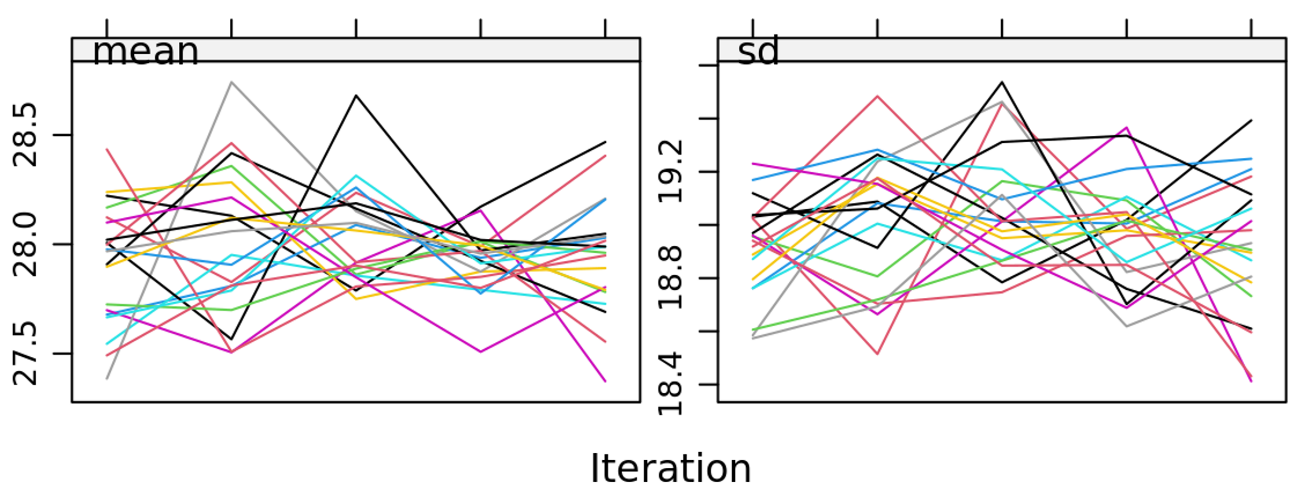
**

**B. Density plot**

**
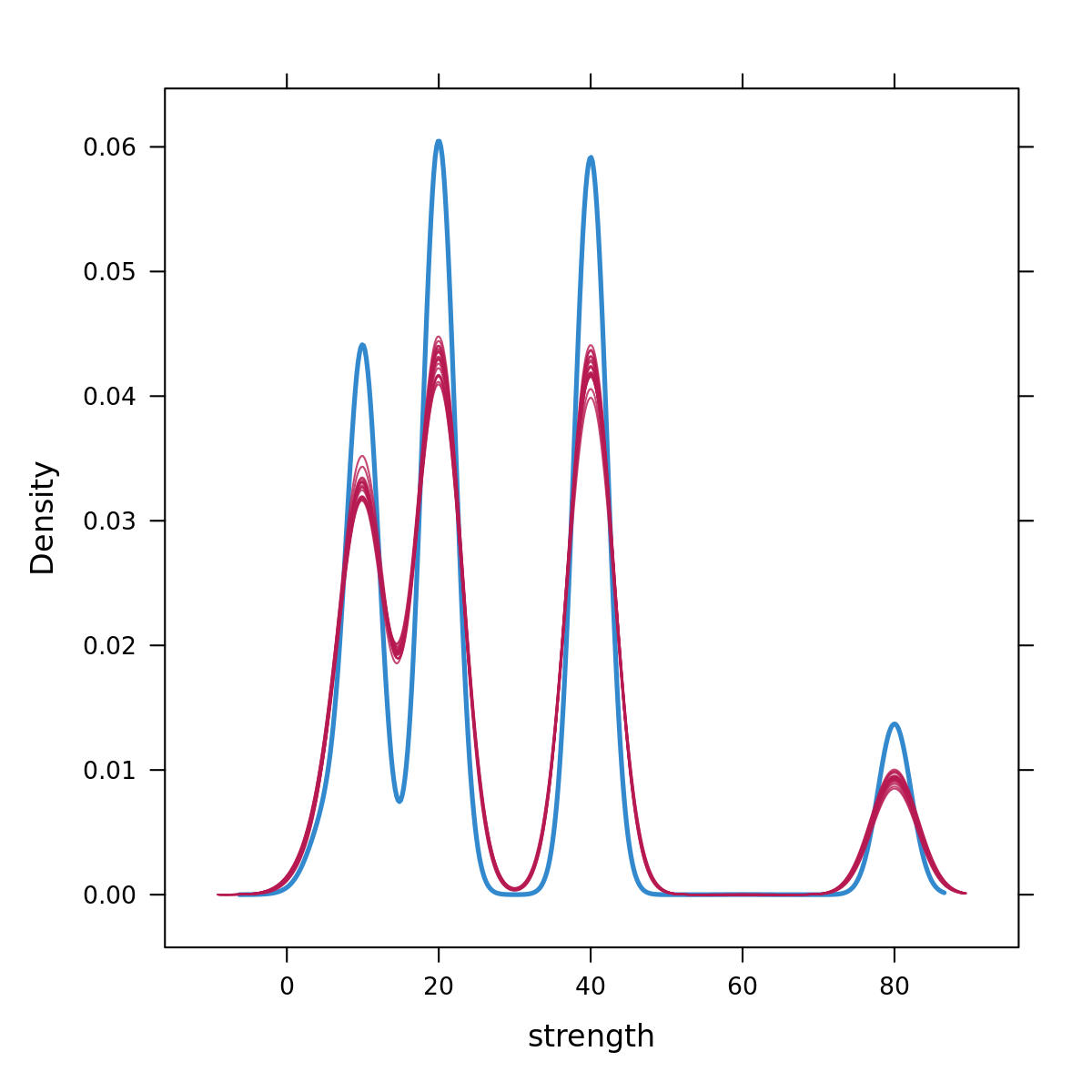
**

**Figure S4. Imputation assessment for statin strength.** The convergence plot (**Panel A**) shows multiple streams freely intermingling without distinct trends, indicating that the imputation algorithm reached convergence. The density plot (**Panel B**) displays overlapping density curves from various iterations (blue: observed; red: imputed) showing the consistency of imputed statin strength values.

**
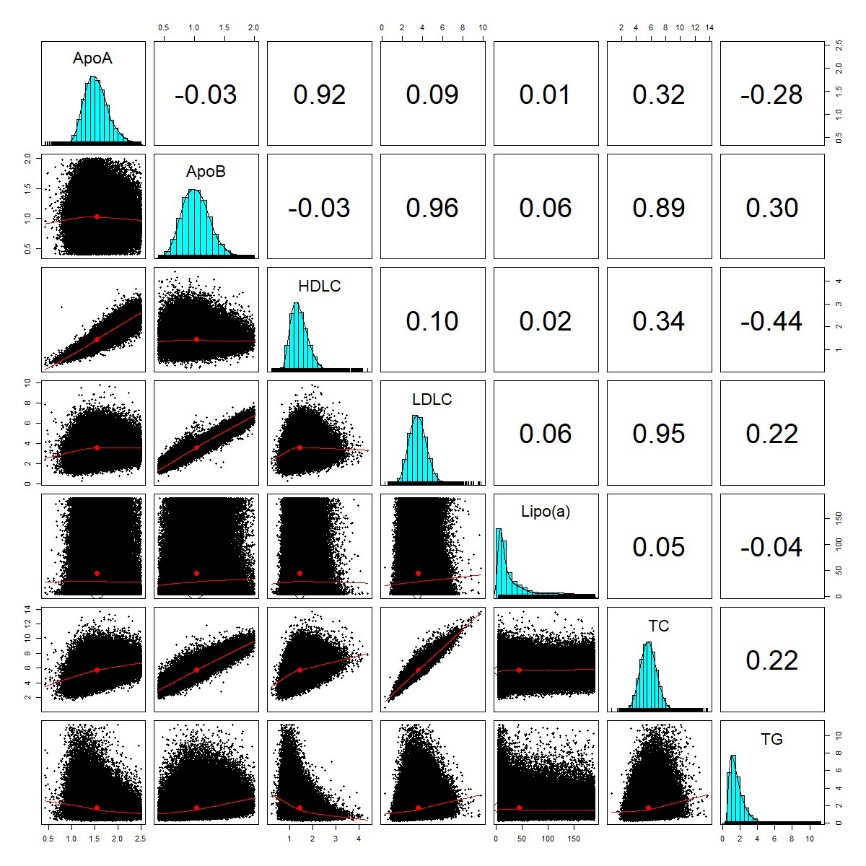
**

**Figure S5. An enhanced scatterplot matrix showing correlations between the lipid biomarkers in the UK Biobank Baseline Analysis (16.5% on statins).** This figure presents a correlation matrix and pairwise scatter plots for key lipid biomarkers: Apolipoprotein A (ApoA), Apolipoprotein B (ApoB), HDL cholesterol (HDLC), LDL cholesterol (LDLC), lipoprotein A (Lipo(a)), total cholesterol (TC), and triglycerides (TG). The upper triangles show Pearson correlation coefficients between the biomarkers, the diagonals display the distribution of each biomarker, and the lower triangles shows scatter plots with locally weighted smoothing (red lines) to depict relationships between the variables.

**A. UK Biobank (pre-statin measurements)**

**
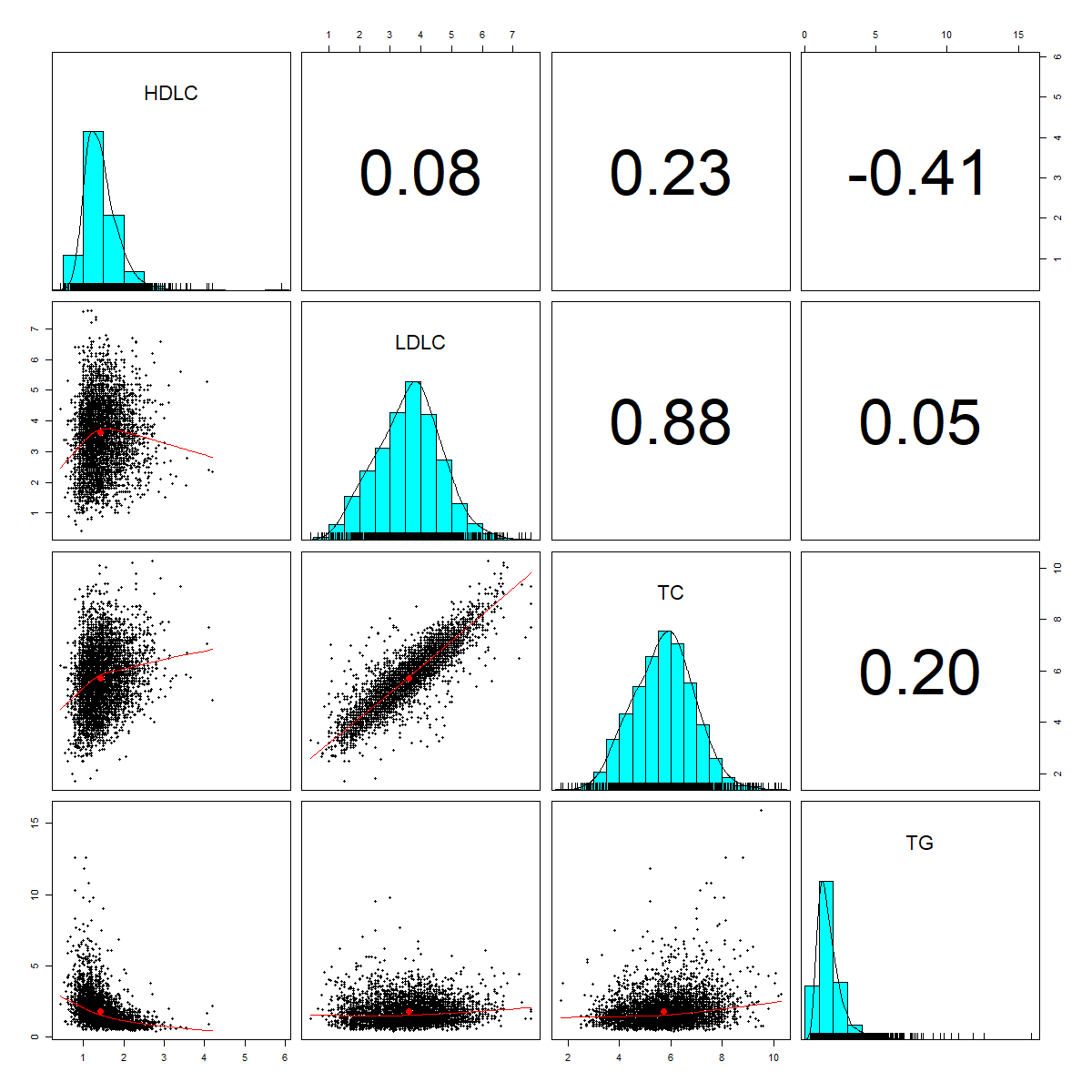
**

**B. UK Biobank (net changes)**

**
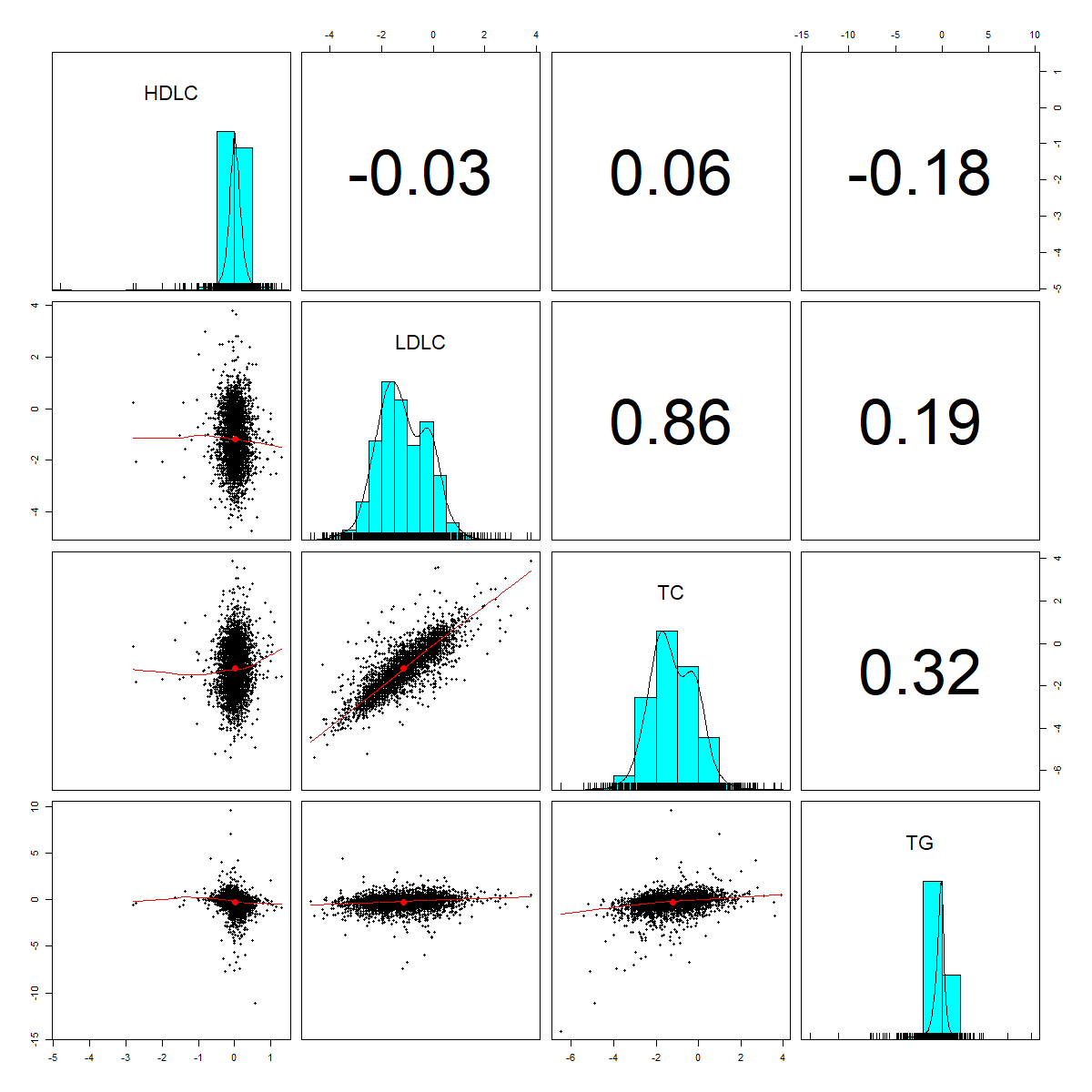
**

**C. All of Us (pre-statin measurements)**

**
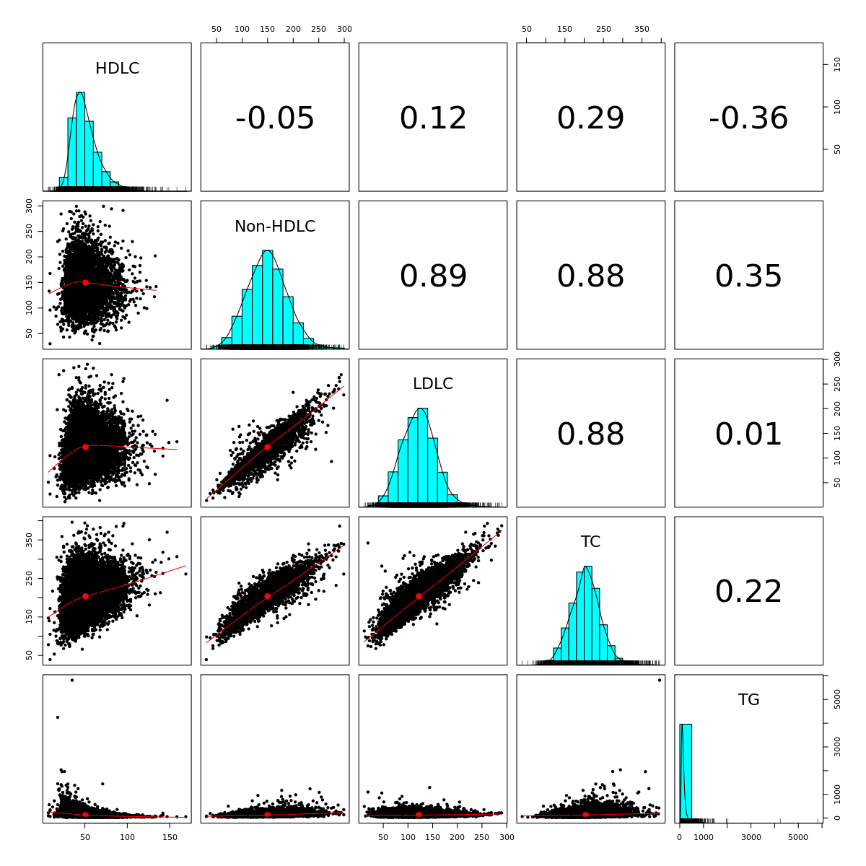
**

**D. All of Us (net changes)**

**
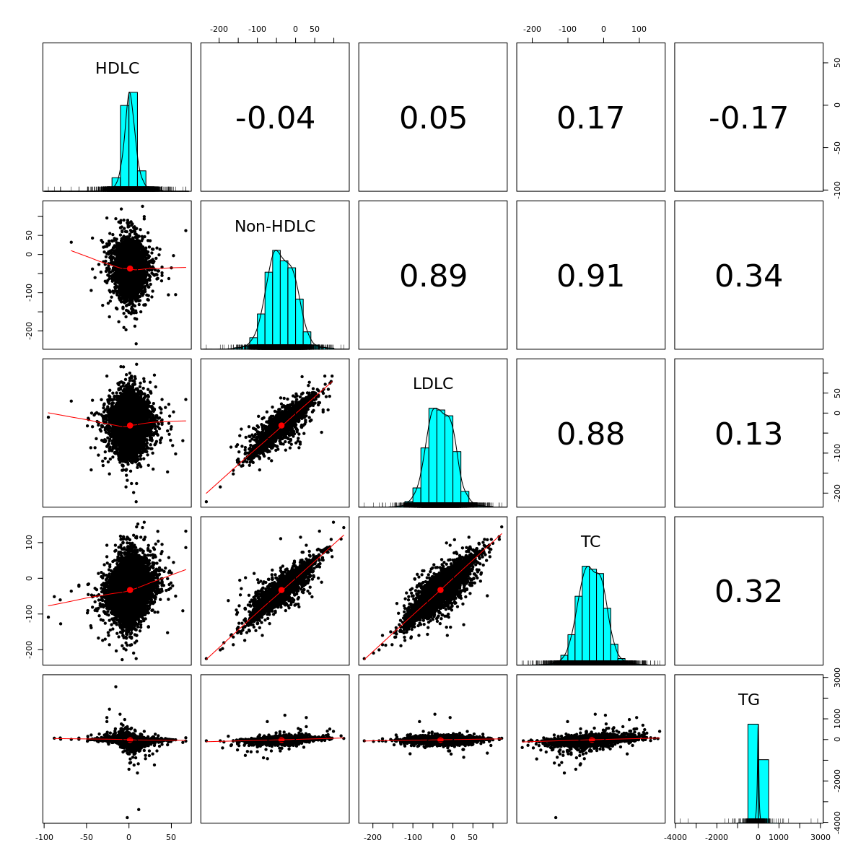
**

**Figure S6. An enhanced scatterplot matrix showing correlations between the lipid biomarkers (electronic health records). A.** UK Biobank pre-statin measurements. **B.** UK Biobank net changes. **C.** All of Us pre-statin measurements. **D.** All of Us net changes. This figure shows a correlation matrix and pairwise scatter plots for key lipid biomarkers: HDL cholesterol (HDLC), LDL cholesterol (LDLC), non-HDLC, total cholesterol (TC), and triglycerides (TG). The upper triangles show Pearson correlation coefficients between the biomarkers, the diagonals display the distribution of each biomarker, and the lower triangles shows scatter plots with locally weighted smoothing (red lines) to depict relationships between the variables.

**A. UK Biobank**


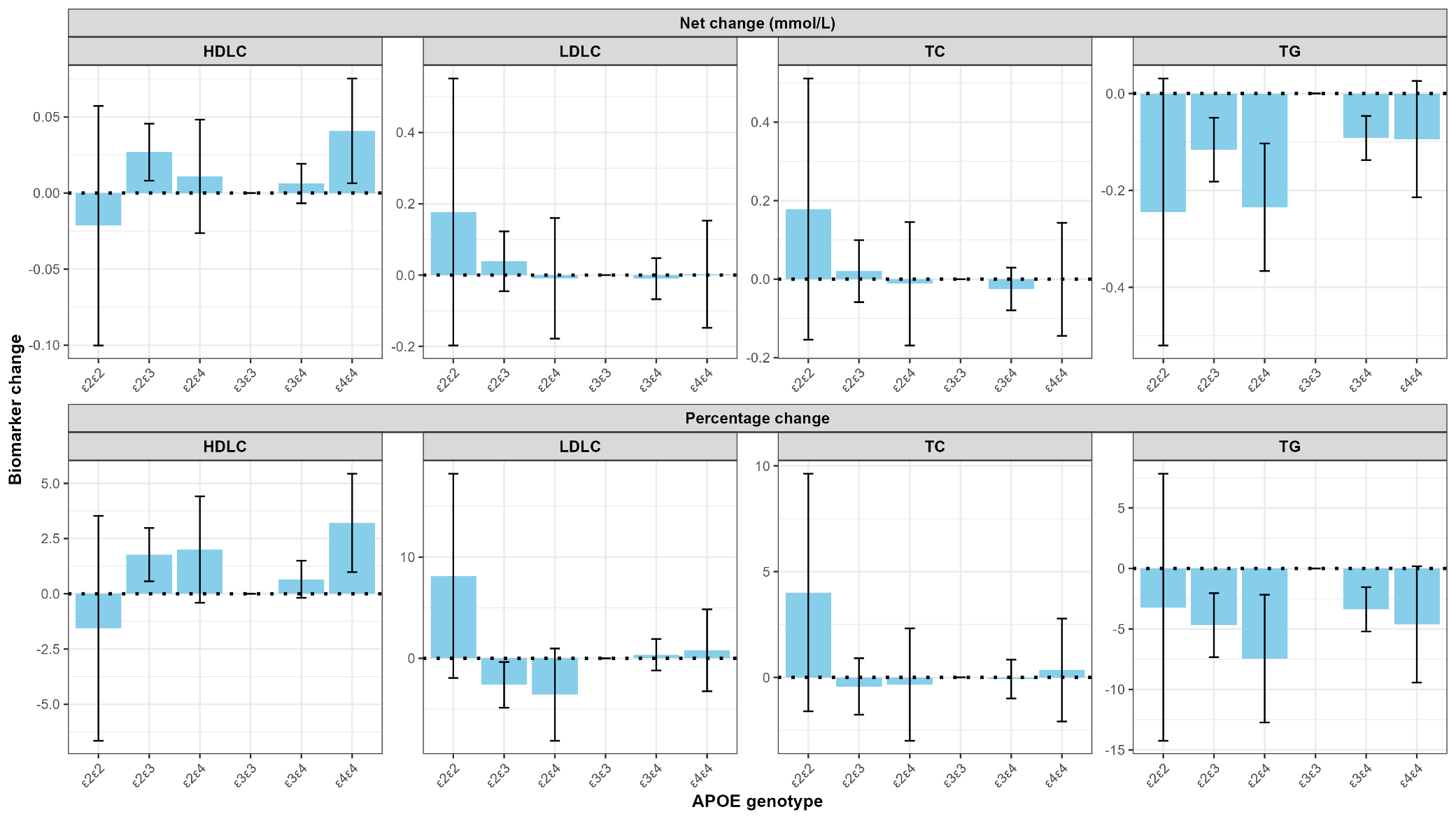


***P = 0.024 P = 0.852 P = 0.775 P < 0.001***

***P = 0.003 P = 0.049 P = 0.764 P < 0.001***

**B. All of Us**

**
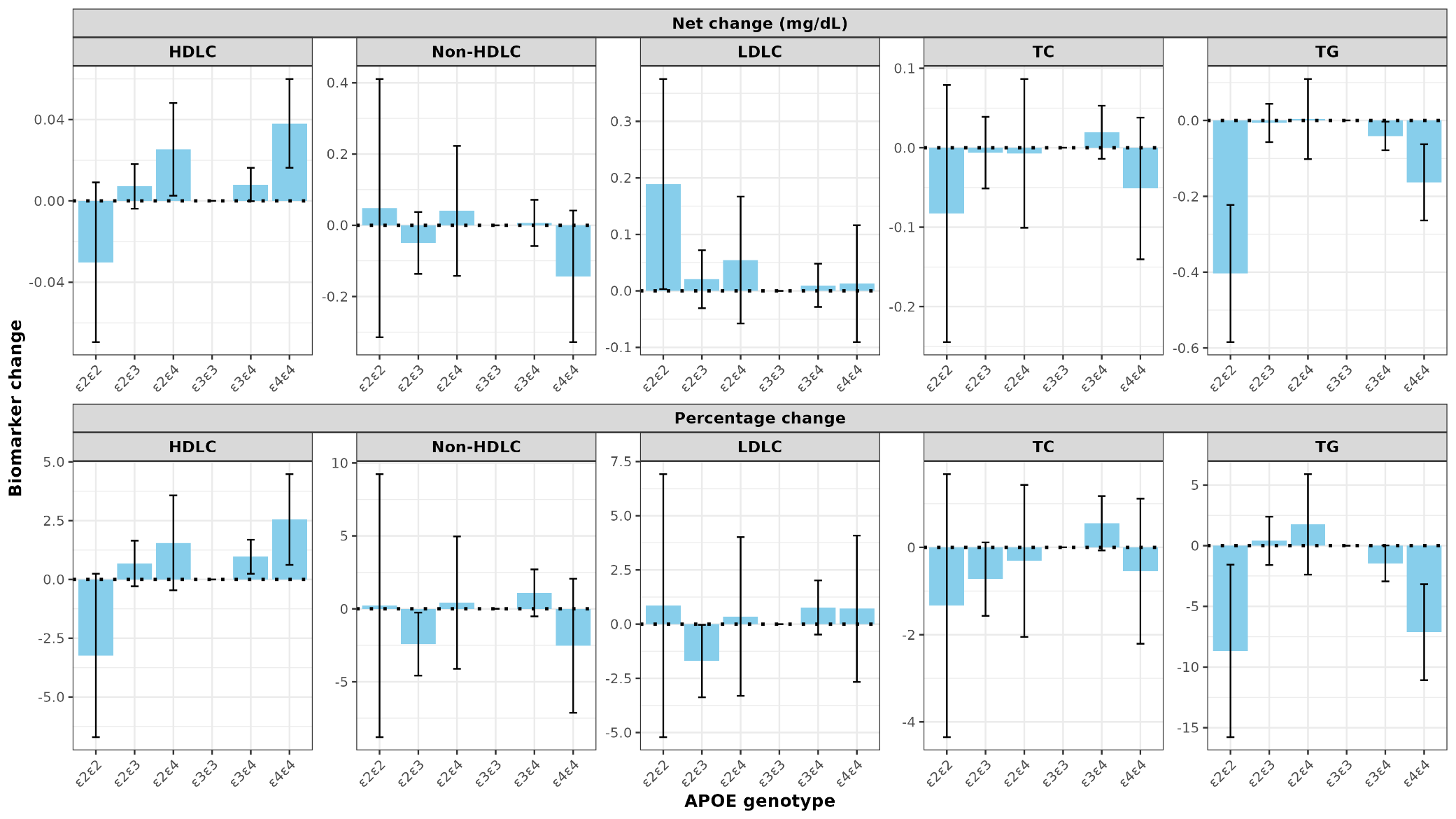
**

***P < 0.001 P = 0.546 P = 0.387 P = 0.520 P < 0.001***

***P = 0.003 P = 0.096 P = 0.223 P = 0.113 P < 0.001***

**Figure S7. Net and percentage changes in lipid biomarkers stratified by *APOE* genotype.** **A.** UK Biobank. **B.** All of Us Program. The top row displays net changes, and the bottom row shows percentage changes. For HDLC (increase beneficial), positive values indicate more benefit with statins relative to the *ε3ε3* genotype, while for other biomarkers (reduction beneficial), negative values indicate more benefit. Error bars represent 95% confidence intervals. APOE = Apolipoprotein E, HDLC = high-density lipid cholesterol, LDLC = low-density lipid cholesterol, TC = Total cholesterol, TG = triglycerides.

**A. UK Biobank**


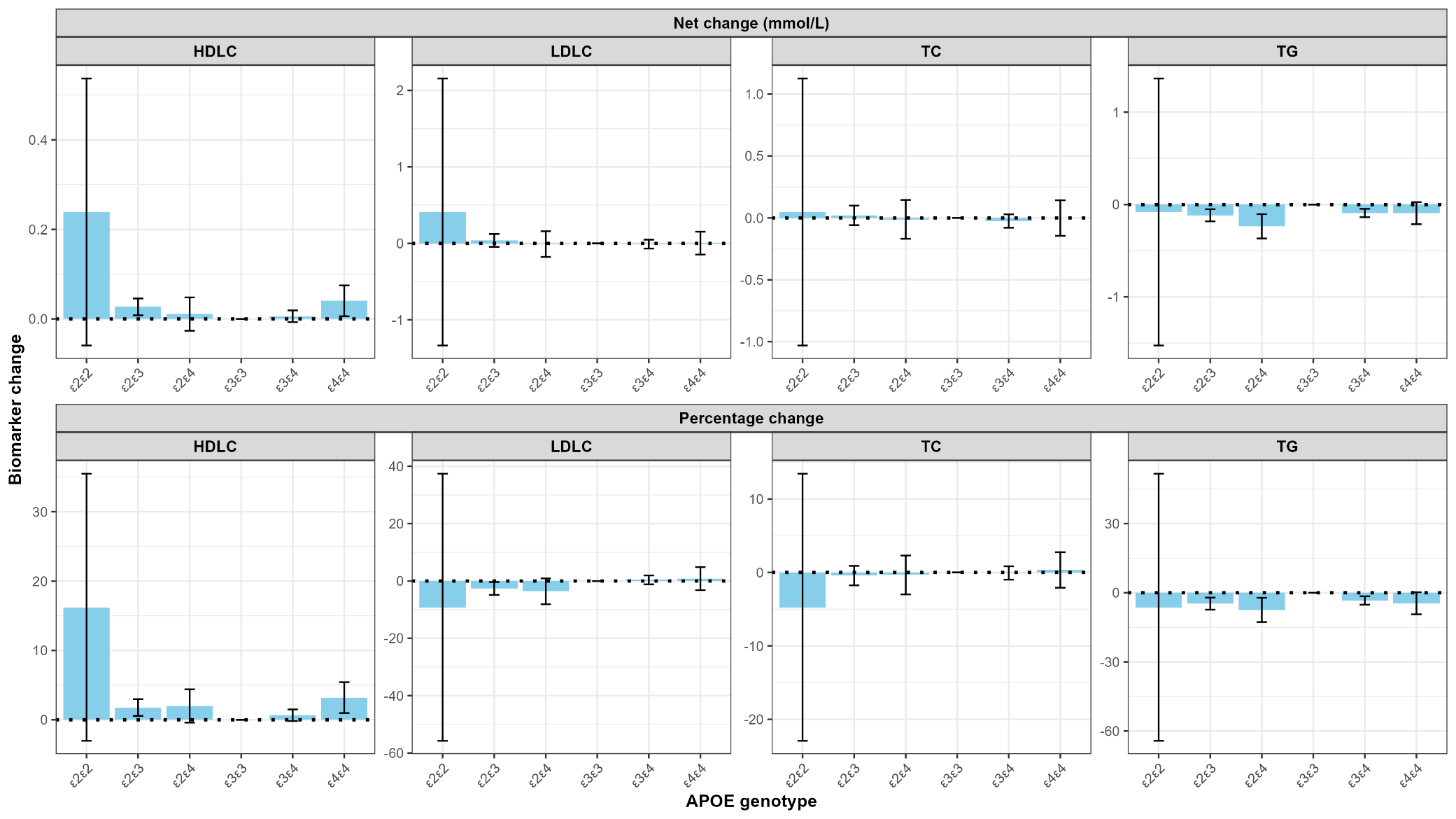


***P = 0.012 P = 0.932 P = 0.929 P < 0.001***

***P = 0.002 P = 0.112 P = 0.976 P < 0.001***

**B. All of Us**

**
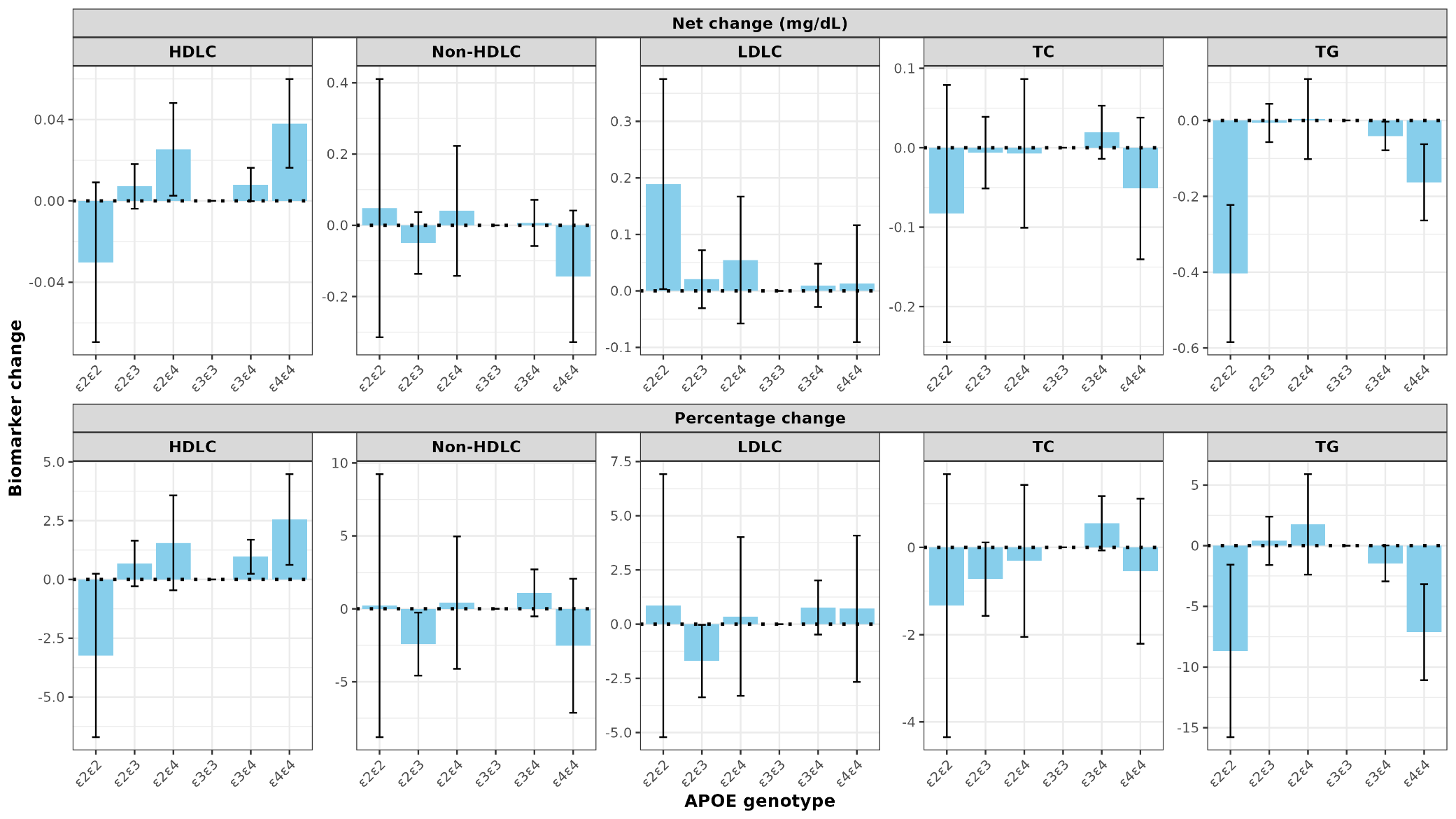
**

***P = 0.001 P = 0.468 P = 0.908 P = 0.375 P < 0.001***

***P = 0.011 P = 0.062 P = 0.217 P = 0.091 P < 0.001***

**Figure S8. Net and percentage changes in lipid biomarkers stratified by *APOE* genotype with dysbetalipoproteinemia individuals excluded. A.** UK Biobank. **B.** All of Us Program. The top and bottom rows respectively show net and percentage changes. For HDLC (increase beneficial), positive values indicate more benefit with statins relative to *ε3ε3*, while for other biomarkers (reduction beneficial), negative values indicate more benefit. Error bars represent 95% confidence intervals. *APOE* = Apolipoprotein E, HDLC = high-density lipid cholesterol, LDLC = low-density lipid cholesterol, TC = Total cholesterol, TG = triglycerides.

**A. UK Biobank**


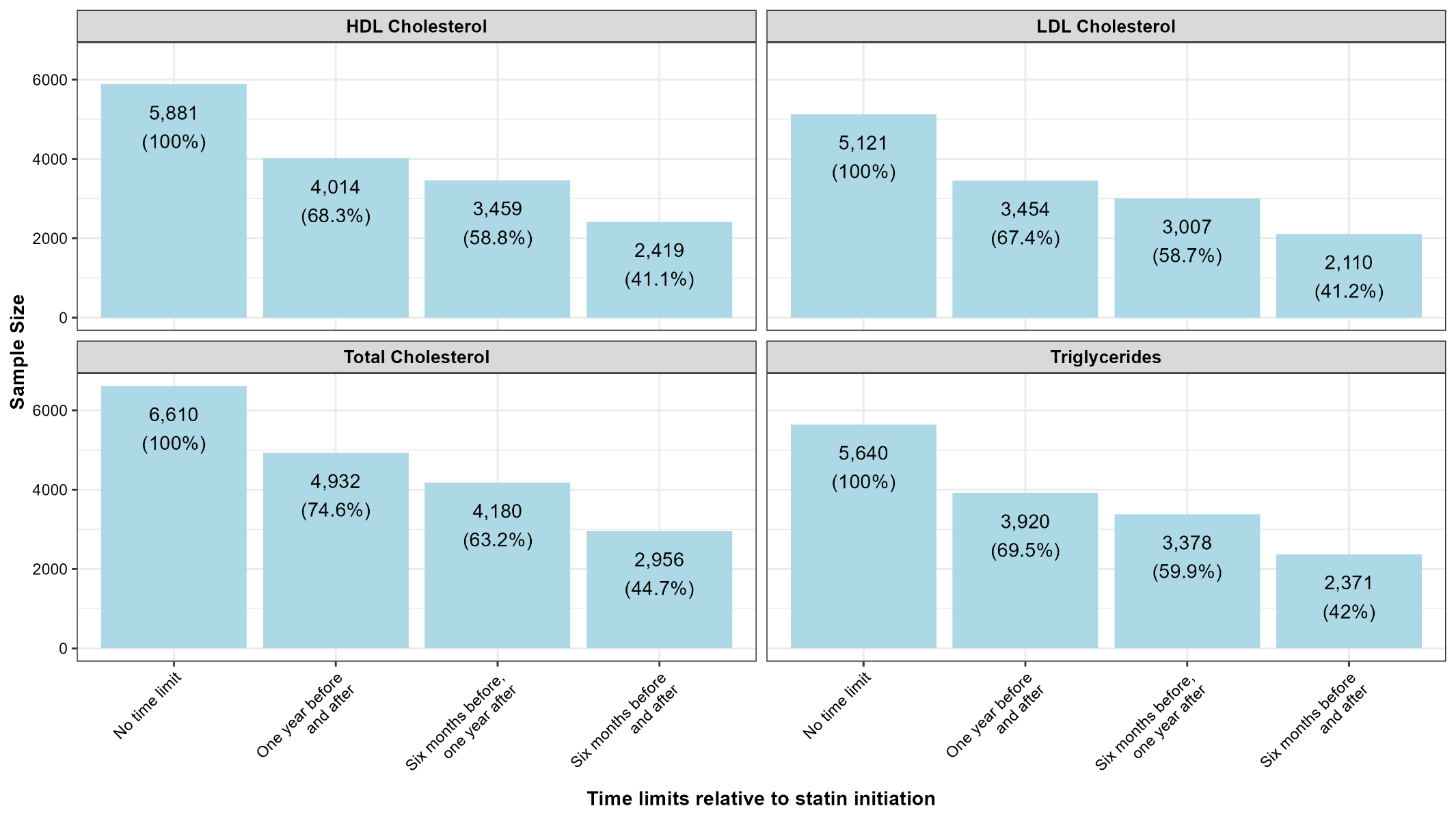


**B. All of Us program**


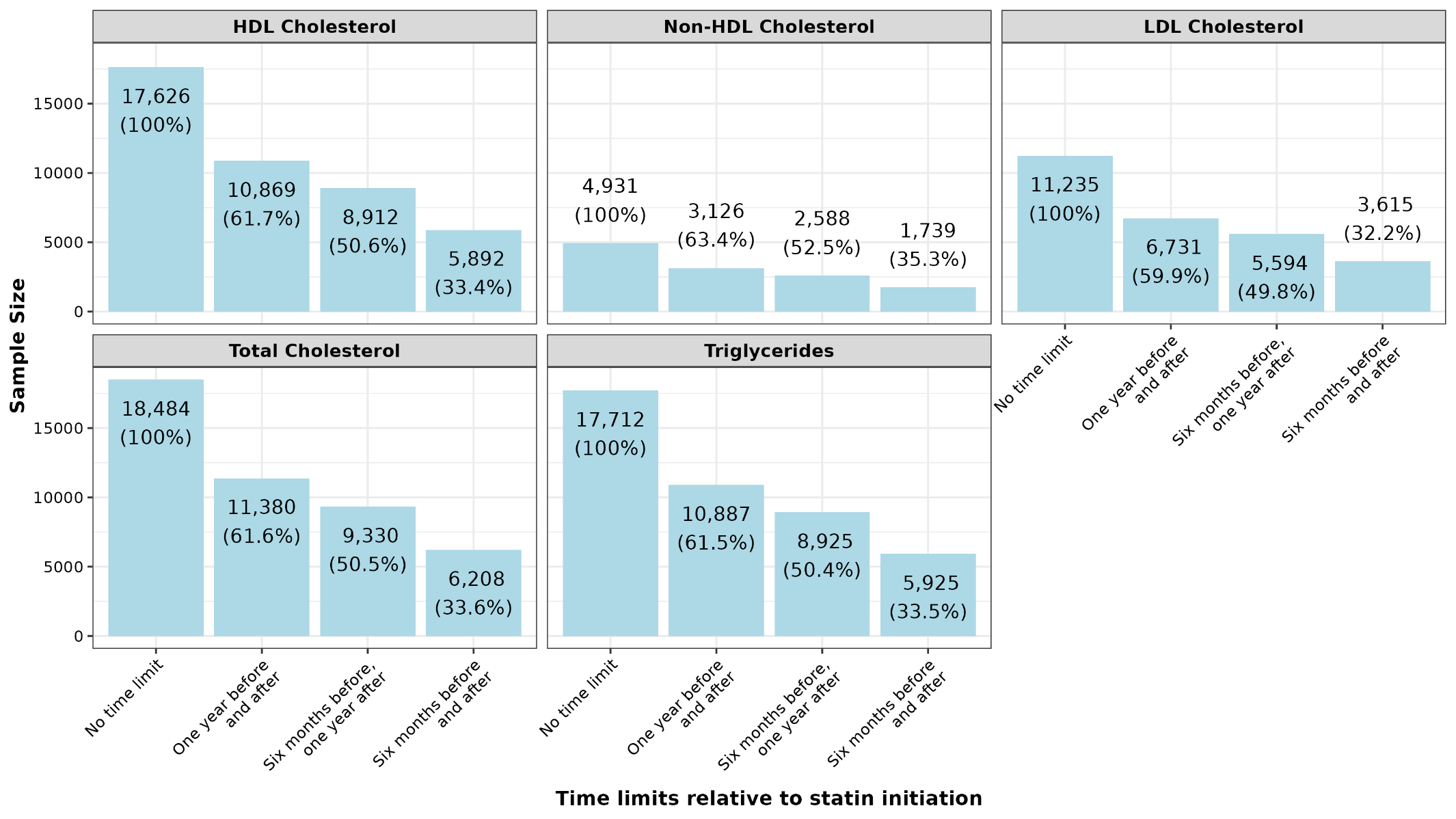


**Figure S9. Sample size across different time limits. A.** UK Biobank. **B.** All of Us program. HDL = high-density lipoprotein, LDL = low-density lipoprotein.

**A. UK Biobank**


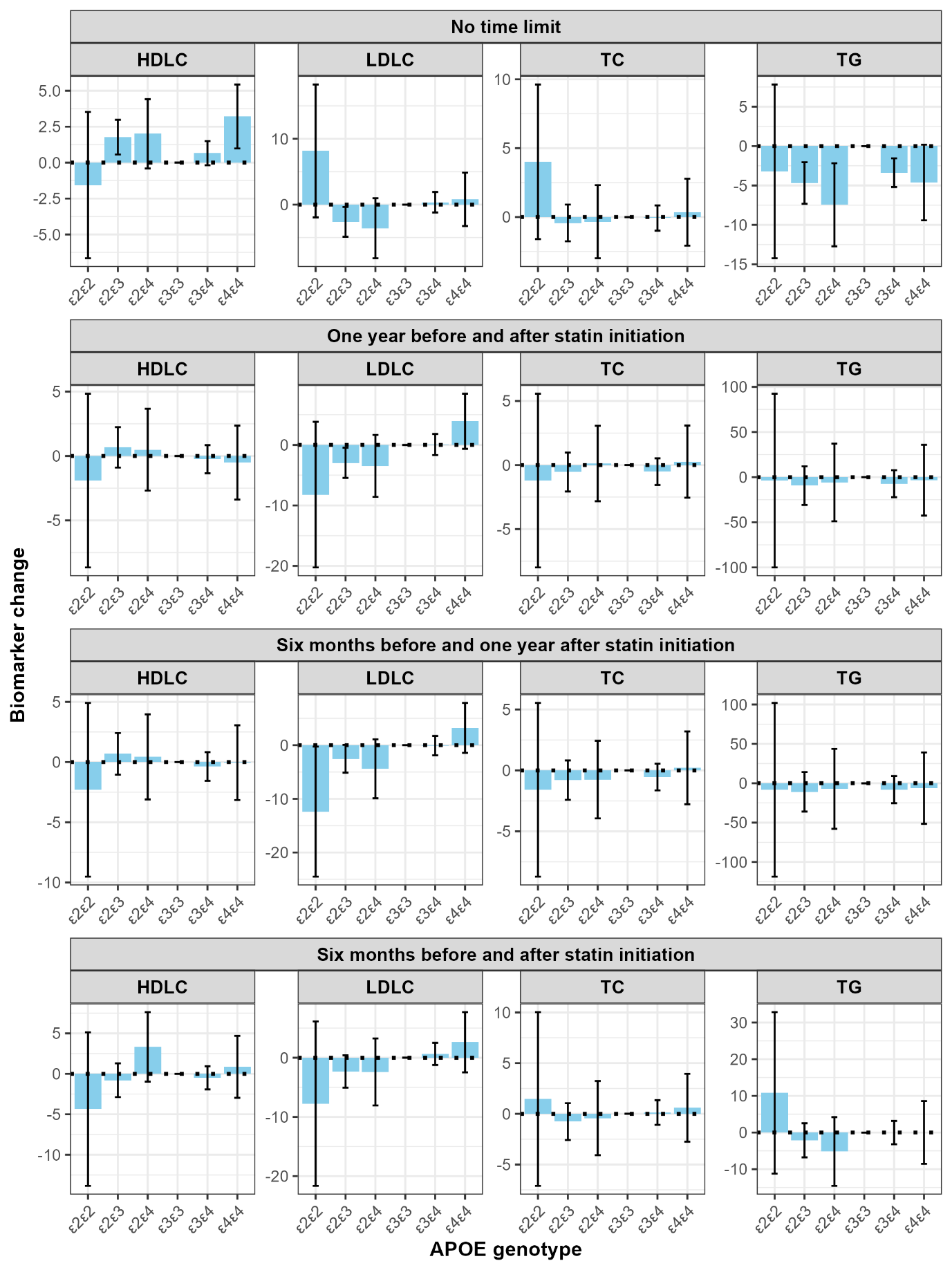


***P = 0.003 P = 0.049 P = 0.764 P < 0.001***

***P = 0.896 P = 0.030 P = 0.920 P = 0.928***

***P = 0.893 P = 0.033 P = 0.865 P = 0.932***

***P = 0.474 P = 0.229 P = 0.951 P = 0.714***

**B. All of Us**

**
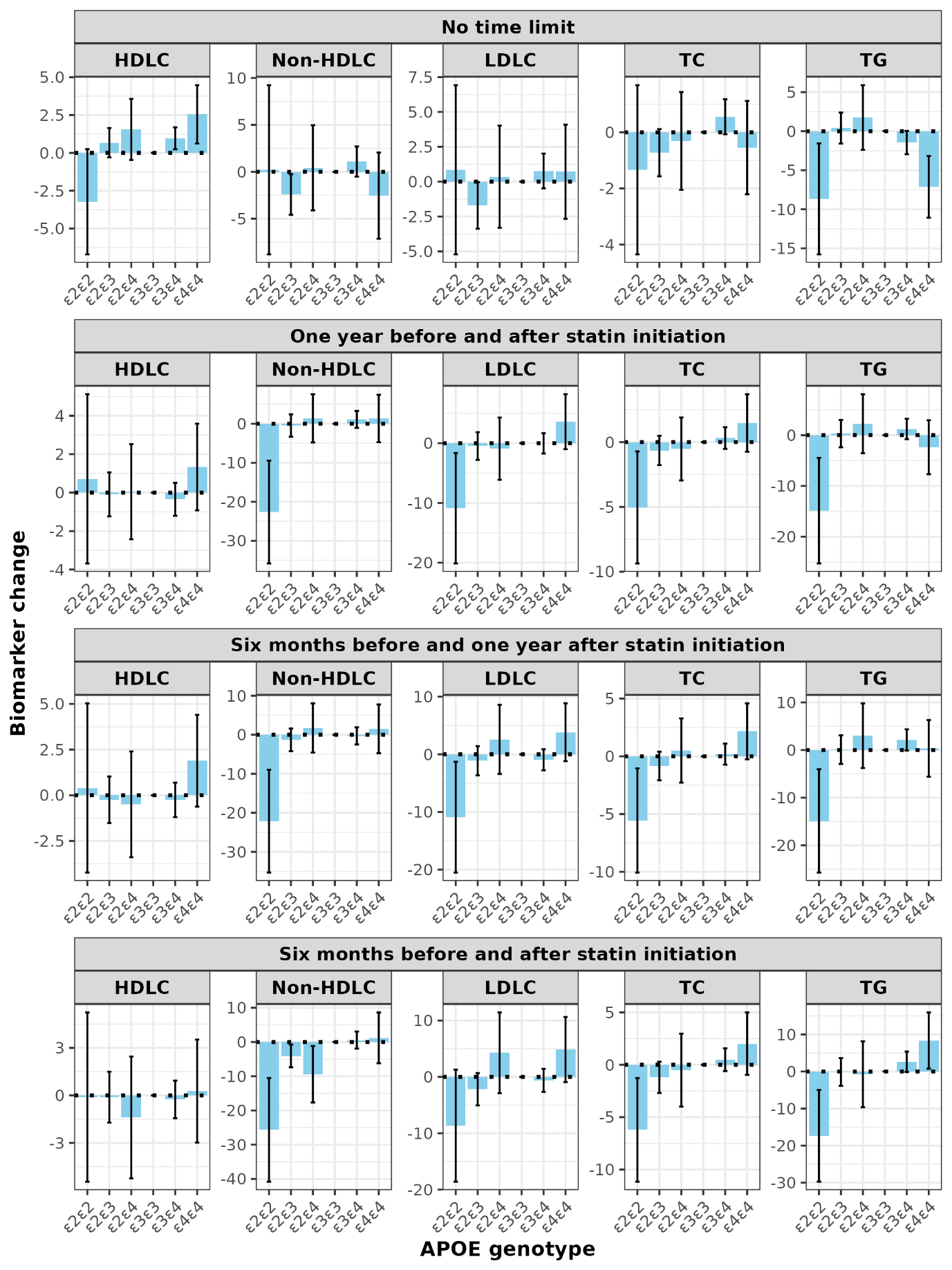
**

***P = 0.003 P = 0.096 P = 0.223 P = 0.113 P < 0.001***

***P = 0.798 P = 0.022 P = 0.151 P = 0.094 P = 0.049***

***P = 0.711 P = 0.033 P = 0.079 P = 0.042 P = 0.035***

***P = 0.983 P < 0.001 P = 0.079 P = 0.034 P = 0.007***

**Figure S10. Percentage changes in lipid biomarkers stratified by *APOE* genotype and the different time limits.** **A.** UK Biobank. **B.** All of Us Program. For HDLC (increase beneficial), positive values indicate more benefit with statins relative to the *ε3ε3* genotype, while for other biomarkers (reduction beneficial), negative values indicate more benefit. Error bars represent 95% confidence intervals. *APOE* = Apolipoprotein E, HDLC = high-density lipid cholesterol, LDLC = low-density lipid cholesterol, TC = Total cholesterol, TG = triglycerides.


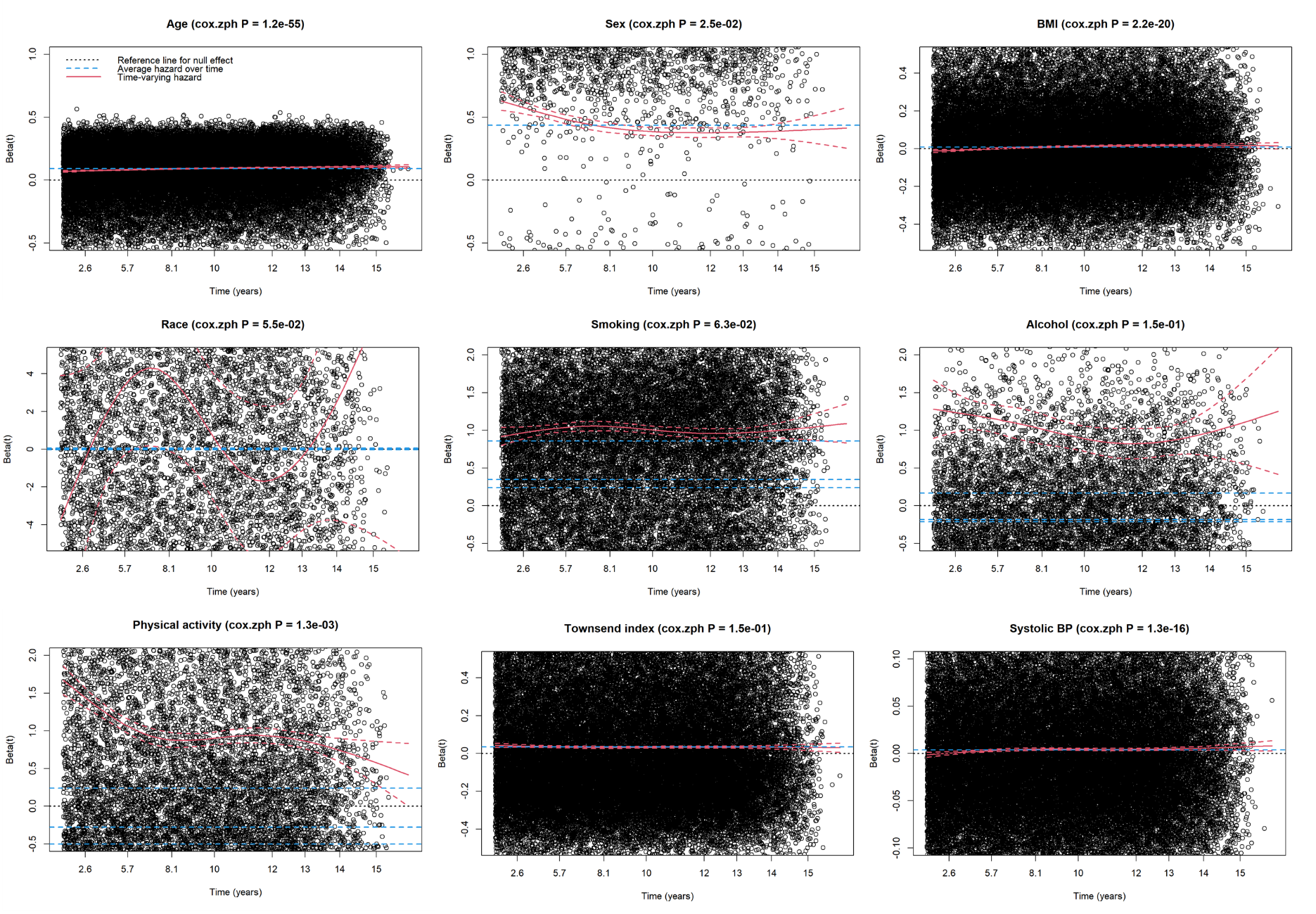


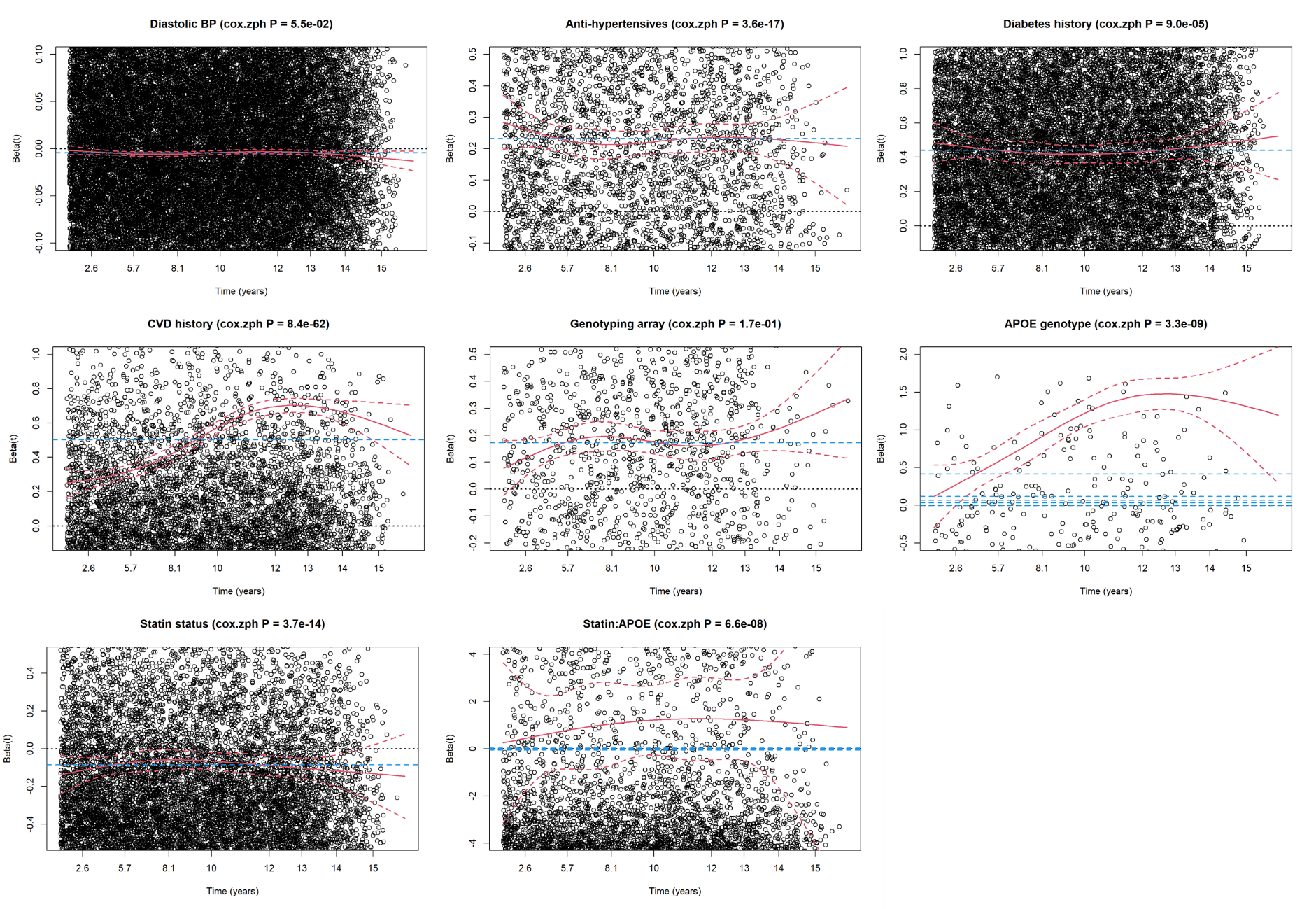


**Figure S11. Results of the proportional hazards assumption test for the analysis of all-cause death in the UK Biobank Baseline Analysis.** *APOE* = Apolipoprotein E, BMI = body mass index, CVD = cardiovascular disease.

**Supplementary References**

1. Moore CM, Jacobson SA, Fingerlin TE. Power and Sample Size Calculations for Genetic Association Studies in the Presence of Genetic Model Misspecification. *Hum Hered* **84** 256-271. (2019)

2. Li M, Zhao JV, Kwok MK, Schooling CM. Age and sex specific effects of APOE genotypes on ischemic heart disease and its risk factors in the UK Biobank. *Sci Rep* **11** 9229. (2021)

3. Qiu W, Chavarro J, Lazarus R, Rosner B, Ma J. powerSurvEpi: Power and Sample Size Calculation for Survival Analysis of Epidemiological Studies. R package version 0.1.3. 2021. pp. 1-61.

4. Asiimwe IG, Gebru T, Jorgensen AL, Pirmohamed M, Multimorbidity Mechanism and Therapeutic Research Collaborative. APOE genotype and the effect of statins: a systematic review and meta-analysis. *medRvix* 1-21. (2024)

5. Mach F*, et al.* 2019 ESC/EAS Guidelines for the management of dyslipidaemias: lipid modification to reduce cardiovascular risk. *Eur Heart J* **41** 111-188. (2020)

6. Grundy SM*, et al.* 2018 AHA/ACC/AACVPR/AAPA/ABC/ACPM/ADA/AGS/APhA/ASPC/NLA/PCNA Guideline on the Management of Blood Cholesterol: Executive Summary: A Report of the American College of Cardiology/American Heart Association Task Force on Clinical Practice Guidelines. *J Am Coll Cardiol* **73** 3168-3209. (2019)

7. Reiner Z. Managing the residual cardiovascular disease risk associated with HDL-cholesterol and triglycerides in statin-treated patients: a clinical update. *Nutr Metab Cardiovasc Dis* **23** 799-807. (2013)

8. Barter PJ, Brandrup-Wognsen G, Palmer MK, Nicholls SJ. Effect of statins on HDL-C: a complex process unrelated to changes in LDL-C: analysis of the VOYAGER Database. *J Lipid Res* **51** 1546-1553. (2010)

9. Sinha M*, et al.* Efficacy of Traditional Anti-lipidemic Drugs in Lowering Lipoprotein(a) Levels: A Systematic Review. *Cureus* **16** e69824. (2024)

10. Baigent C*, et al.* Efficacy and safety of cholesterol-lowering treatment: prospective meta-analysis of data from 90,056 participants in 14 randomised trials of statins. *Lancet* **366** 1267-1278. (2005)
